# Historical photographs revisited: A case study for dating and characterizing recent loss of coral cover on the inshore Great Barrier Reef

**DOI:** 10.1038/srep19285

**Published:** 2016-01-27

**Authors:** Tara R. Clark, Nicole D. Leonard, Jian-xin Zhao, Jon Brodie, Laurence J. McCook, David R. Wachenfeld, Ai Duc Nguyen, Hannah L. Markham, John M. Pandolfi

**Affiliations:** 1 School of Earth Sciences, The University of Queensland, Brisbane QLD 4072 Australia; 2 Centre for Tropical Water and Aquatic Ecosystem Research, James Cook University, Townsville, QLD 4811 Australia; 3 ARC Centre of Excellence in Coral Reef Studies, James Cook University, Townsville, QLD 4811 Australia; 4 Great Barrier Reef Marine Park Authority, 2-68 Flinders Street, PO Box 1379, Townsville, QLD 4810 Australia; 5 ARC Centre of Excellence in Coral Reef Studies, School of Biological Sciences, The University of Queensland, Brisbane QLD 4072 Australia

## Abstract

Long-term data with high-precision chronology are essential to elucidate past ecological changes on coral reefs beyond the period of modern-day monitoring programs. In 2012 we revisited two inshore reefs within the central Great Barrier Reef, where a series of historical photographs document a loss of hard coral cover between c.1890–1994 AD. Here we use an integrated approach that includes high-precision U-Th dating specifically tailored for determining the age of extremely young corals to provide a robust, objective characterisation of ecological transition. The timing of mortality for most of the dead *in situ* corals sampled from the historical photograph locations was found to coincide with major flood events in 1990–1991 at Bramston Reef and 1970 and 2008 at Stone Island. Evidence of some recovery was found at Bramston Reef with living coral genera similar to what was described in c.1890 present in 2012. In contrast, very little sign of coral re-establishment was found at Stone Island suggesting delayed recovery. These results provide a valuable reference point for managers to continue monitoring the recovery (or lack thereof) of coral communities at these reefs.

Monitoring programs within the Great Barrier Reef World Heritage Area (GBRWHA) have recently revealed dramatic losses in coral cover on the mid-shelf and offshore reefs^1^. Declining water quality as a result of rapid coastal development since European settlement (c.1850) in conjunction with crown-of-thorns starfish outbreaks and coral bleaching, is believed to have also resulted in a decline in coral cover and changes in community composition on inshore reefs^2^,^3^,^4^,^5^. This rather austere outlook is further exacerbated by the Great Barrier Reef (GBR) contentiously being argued as *not* the best managed reef in the world, with delays in water quality improvement programs and the threat of increased port development along the Queensland coastline^6^,^7^. The seriousness of the current state of the GBR has manifested in its potential delisting from World Heritage to World Heritage in Danger by the United Nations Educational, Scientific and Cultural Organisation (UNESCO).

Yet there is still considerable disagreement regarding the overall health of coral reefs on the GBR^8^,^9^,^10^,^11^ and the link between reef decline and water-quality change^12^. Key to the debate is the absence of systematic monitoring studies over relevant timescales. To resolve this discord, detailed baseline studies based on a high-precision chronology long enough to capture a reef’s natural range of variability are essential in understanding the timing, rate and spatial variability of the loss of reef-building coral communities and whether their losses can be attributed to longer-term geomorphological or climatic processes, as well as recent human and environmental impacts^13^,^14^.

Fortunately, palaeo-ecological and -environmental studies can provide a means to understand centennial to millennial-scale ecological changes on inshore reefs and their response to recent environmental change e.g.^3^,^15^,^16^; however, these are often spatially limited and strongly influenced by time averaging and taphonomic processes^17^,^18^. Nevertheless, increased replication and high-resolution isotopic dating is helping to overcome these limitations.

Comparisons between historical and modern photographs can also provide a powerful visual tool to assess recent changes in environmental condition following European settlement. For instance, using anecdotal comparisons of a collection of historical photographs taken from c.1890 onwards of reef flat and reef crest communities from 14 locations, Wachenfeld^19^ demonstrated that four locations showed significant changes in benthic community structure including a marked decline in hard coral cover. However, historical photographs are qualitative not quantitative, represent an incomplete temporal record and the original photographers were likely to be biased towards more picturesque areas of the reef. Such a comparative approach has significant limitations as definitive proof of declines on inshore reefs of the GBR^19^. Importantly, the photographic comparisons provide minimal constraint on the timing of the changes observed and subsequent linkages to possible drivers. The integration of historical photographic comparisons with high-precision U-Th dating can help to place these changes in an accurate chronological timeframe; markedly increasing their interpretive value to managers.

In this study we revisit Bramston Reef and Stone Island; two of the historical photograph locations in Wachenfeld^19^ that have shown an apparent decline in coral cover over the past 100 years (Fig. 1). Using standard benthic transects and the highly precise U-Th dating technique, we determine the timing of coral mortality, and provide an important ecological baseline with which to judge the recovery or decline of modern coral communities. This approach can be applied to other sites having undergone changes in coral community structure in order to provide a broad scale understanding of changes on the GBR.

## Results

### Modern benthic composition

To assess the recovery, or lack thereof, of coral communities, the same sites at Bramston Reef and Stone Island photographed in 1994 were located using the bearings and geographic features described by Wachenfeld^19^ (1994 surveys predate the availability of hand held GPS). Bramston Reef was revisited at a tide of 0.18 m lowest astronomical tide (LAT) on 31 July 2012 and the same area also identified by the presence of the conspicuously large, dead *in situ* faviids photographed in 1994. The first photograph was taken looking south-east over Bramston Reef with Gloucester Island and Cape Gloucester as the landmark feature in the background (Fig. 2e; Supplementary Table S1), while the second photograph looks north-east towards Stone Island (Fig. 2f; Supplementary Table S1). In both photographs, a large number of dead faviids dominate the benthos in the foreground of the images. The dead faviids extend for several hundred metres alongshore and form a zone ~70 m wide along the reef edge before transitioning into a living *Montipora* sp. dominated coral community towards the mainland (Fig. 3a). The dead coral surfaces and sediment were mostly covered by turf or macroalgae. Living faviid colonies are also present, with the majority being small (<20 cm). Larger colonies were found (Supplementary Fig. S3e), however, they were much lower in abundance than their non-living counterparts. In Supplementary Figure S3, tabular *Acropora* colonies can also be seen. Benthic surveys conducted at Bramston Reef at the same location as the 1994 photographs revealed mean live coral cover to be 7.0 ± 4.7% [±1 standard deviation (sd)]. Living coral genera included *Acropora, Goniastrea, Montipora, Goniopora, Lobophyllia, Favites, Turbinaria, Pocillopora* and *Favia*. Despite a high abundance of dead faviid colonies, mean dead coral cover was low (7.4 ± 6.4%) as most surfaces were covered by algae, accounting for 45 ± 9% of the benthic composition [Fig f4](Fig. 5a).

In addition to the 1994 photographs, the c.1890 photographs taken by Saville-Kent^20^ were also used locate the same sites depicted in the images of Stone Island. The first photograph was taken at a low tide of 0.25 mLAT on the 30 July 2012 looking south-east with Gloucester Island and Cape Gloucester as the landmark feature in the background (Fig. 4f; Supplementary Table S1). At the reef crest, the substrate reflected a flattened zone of branching coral rubble and algae, occasionally interspersed by dead tabular *Acropora* and sub-massive colonies believed to have died *in situ*. Here, mean live coral cover was extremely low (0.09 ± 0.12%) with only *Acropora* captured in the analysis (Fig. 5b). Other coral genera that were present along the transects included *Cyphastrea*, *Pocillopora*, *Goniastrea*, *Platygyra* and *Favia*. The second photograph was taken the following day at 0.18 mLAT on 31 July 2012 looking south-east with Cape Gloucester predominately featuring in the background of the image (Fig. 4e; Supplementary Table S1).

### Geomorphology

The uppermost limit of open-water coral growth on the GBR approximates the mean low water spring (MLWS) tide level plus or minus 25 cm due to inter-annual variation in tidal behavior, exposure to wave action, and the varying tolerance of different coral species to exposure, with some faviid corals (including *Goniastrea* and *Platygyra* sp.) able to grow to levels slightly higher than MLWS height^21^,^22^. As this could explain the apparently degraded state of Bramston Reef and Stone Island, elevation surveys were conducted to obtain an understanding of reef flat topography and height of *in situ* live/dead coral colonies relative to MLWS height.

At Bramston Reef, we found the reef crest to be below MLWS height (0.67 mLAT) at the same location as the 1994 photographs, ranging between −0.31 to 0.49 mLAT and progressing through three distinct zones (Fig. 3a). From the shore to the start of the elevation transect (Zone 1, 0–1,000 m), reef substrate was mostly covered by sand and mud, with the height of a half-buried microatoll determined to be at 0.64 mLAT, This zone then transitioned into a living *Montipora* sp. dominated community (Zone 2, ~1060–1,190 m) with an average elevation of 0.26 ± 0.09 mLAT. Towards the edge of the reef (Zone 3, ~1,190–1,270 m from the shore), benthic composition closely resembled what can be seen in the historical photographs with numerous large dead faviids creating an undulating topography with substrate height being on average 0.08 ± 0.21 (1sd) mLAT (*N* = 6). The elevation of six massive dead faviids from this zone was reported to be within error of the MLWS level, ranging in height between 0.57 to 0.66 mLAT [average 0.60 ± 0.03 (1sd)] (Fig. 3a). Large living faviid colonies were also occasionally found growing *above* MLWS tide height at 0.70 mLAT (Fig. 3a; Supplementary Fig. S3e).

At Stone Island, average elevation of the reef substrate was higher than MLWS height at the start of the transect closest to shore [0.90 ± 0.09 mLAT (*N* = 3)] where large fossil microatolls up to 2.6 m in diameter characterized the benthos. The reef flat then abruptly stepped down to 0.15 ± 0.22 mLAT (*N* = 9) between ~20–240 m and was largely still submerged at low tide. Here, living tabular *Acropora* sp. and *Porites* sp. colonies were occasionally found. Towards the reef crest and at the location of the historical photographs, substrate height increased to 0.23 ± 0.11 mLAT (*N* = 2) at a distance of 240–290 m from shore (Fig. 3b).

### Timing of mortality

High-precision U-Th dates of 17 randomly collected dead *in situ* faviid colonies (identified as *Goniastrea aspera* and *Platygyra verweyi*) that predominately feature in the 1994 photographs of Bramston Reef, revealed the timing of mortality to have mostly occurred between 1984.6 ± 4.7 and 1993 ± 10, analytically almost indistinguishable from a weighted mean of 1990.4 ± 1.3 (2-sigma) (Fig. 6f). Only three samples (BRS1T1.1, BRS1T2.1, and BRS1T4.4) define significantly different mortality ages of 1853.0 ± 2.8, 1968.2 ± 4.4 and 2005.6 ± 7.2, respectively. All U-Th data are considered reliable with uranium concentrations (ranging between 2.4–2.9 ppm) and ^234^U/^238^U activity ratios (ranging between 1.143–1.150) similar to that of modern corals reported elsewhere on the GBR^23^,^24^,^25^. Rigorous cleaning of the coral samples removed most of the detrital ^232^Th, with samples containing on average 1.34 ppb (range 0.30 to 4.57 ppb) and resulted in uncorrected ^230^Th age errors of between 0.6 and 1.8 years (Supplementary Table S4).

Two dead *in situ* colonies sampled at the reef crest of Stone Island were identified as *Acropora* sp. and *Porites lobata* and were found to be more than 6,000 years old (SIS1T2.2 and SIS1T2.4). The ^230^Th age data is in agreement with ^14^C ages obtained from fossil microatolls close to the reef edge at Stone Island in an earlier study^26^ and reflect the timing of when most of the reef development took place. The ^230^Th age data from the remaining seven samples (a mixture of tabular *Acropora* sp., *Montipora turgescens*, *Cyphastrea serailia*) cluster around two more recent time periods in the mid-20th [between 1964.1 ± 6.7 and 1977.5 ± 5.6 (N = 3)] and mid-21st [between 2006.4 ± 3.1 and 2007.3 ± 2.9 (N = 4)] Century and overlap in age with those from Bramston Reef (BRS1T1.1 1968 ± 4.4 and BRS1T4.4 2005.6 ± 7.2, respectively). When both the Stone Island and Bramston Reef ^230^Th age data are considered together, the ages return a weighted mean of 1970.4 ± 9.6 (N = 4) and 2007.2 ± 1.4 (N = 5) for each of the two periods. Detrital ^232^Th was slightly higher in the Stone Island samples with average concentrations of 4.73 ppb (range 0.99 to 23.4 ppb), which consequently resulted in larger uncorrected ^230^Th age errors of between 0.6 and 29 years (Supplementary Table S4).

## Discussion

Using a combination of anecdotal, ecological and geochemical techniques, the results of this study provide a robust understanding of coral community change for Bramston Reef and Stone Island. In the late 19th and early 20th Century, historical photographs revealed large and abundant living tabular *Acropora* sp. and massive faviid colonies at Bramston Reef (Fig. S2a,b and Supplementary Fig. S1) and high cover of both plating and branching *Acropora* sp. colonies at Stone Island (Fig. S3a,b). By contrast in 1994, no living *Acropora* colonies were found at either location and the majority of the large faviids that featured so prominently at Bramston Reef in c.1890 were dead, covered in algae and/or mud^19^ (Figs 2c,d and 4c,d). These observations led Wachenfeld^19^ to conclude that a decline in live coral cover had occurred within the area. However, these photographs cannot provide definitive proof that these reefs are in a state of decline as they only offer two (three for Stone Island: c.1890, 1915 and 1994) qualitative ‘snap-shots’ over a ~100 year time period.

In 2012 (eighteen years later), Bramston Reef was still characterized by many large faviid colonies, dead and overgrown by algae and sediment, as well as a large number of small living faviid colonies. Yet there was evidence of some small increase in coral cover, primarily driven by tabular *Acropora* sp. and other genera (Fig. 5a; Supplementary Fig. S3). In addition, living faviid colonies that appeared to be of equal size to their predecessors were also found in 2012, albeit scarce (Supplementary Fig. S3e). At Stone Island, the reef crest was similar to that observed in 1994 with a substrate almost completely devoid of living corals. The timing of the changes observed in the photographs then becomes particularly important not only for understanding the potential drivers behind the coral mortality, but also to reliably assess the current status of the reef by gaining some perspective on the length of time between disturbance and recovery.

U-Th age data obtained from 14 of 17 dead *in situ* faviid colonies sampled from Bramston Reef revealed the timing of mortality to have mostly occurred around 1990.4 ± 1.3 AD, precisely bracketing the time period during which two major events are likely to have caused significant mortality in coral communities. The first event occurred on the 11 December 1989 where an extremely low tide of −0.1 mLAT was reported at the Bowen tidal gauge station; the lowest on record for the period spanning 1986–2013 (Fig. 6b). Similarly low tides of −0.01 and −0.02 mLAT were also reported in the following months of January and February 1990, respectively. With many of the coral colonies at Bramston Reef already growing within error of the MLWS level (the upper-most limit of coral growth; Fig. 3a), this event would have left them aerially exposed for an extended period of time. Such extreme low tide events have been documented to induce coral bleaching and cause high levels of mortality^27^,^28^.

The extreme low tide event was soon followed by two major tropical cyclones, Ivor and Joy, both of which occurred in 1990 (Fig. 6d; Supplementary Table S5). Each initially made landfall as a category 3 and 4 cyclone in the north of Queensland, respectively, yet as they tracked south towards Bramston Reef, both cyclones dissipated into tropical lows causing torrential rain and severe flooding of the central Queensland coastal plain^29^. During cyclone Joy, heavy rain occurred from 26 December 1990 until 4 February 1991, causing major flooding of the Don and Gregory Rivers, and surrounding farming lands of the Don River delta^30^,^31^. At the peak of the flood, river discharge from the Don River exceeded 285,000 megalitres (ML), the highest on record since 1984 when discharge data was first collected, and 90,576 ML for the Gregory River, which was also the highest recorded discharge event based on a 39 year record^31^. During 14–27 January, winds were predominantly northerly^32^ and likely to have pushed the flood plume south into Edgecumbe Bay. Significant increases in dead coral cover also occurred post-cyclone Joy at other nearby inshore reef environments^29^,^33^. Therefore factors associated with major flooding (enhanced turbidity, lowered salinity) may also have been responsible for mortality at Bramston Reef. These events are similar to those experienced in January 1918 as a result of two major cyclones near Mackay. After intense rainfall, a northerly wind drove the freshwater plume (‘a yard in depth’) from the Don River south to Stone Island, resulting in a catastrophic loss of coral cover^34^,^35^.

Although outside the time period that brackets the mortality event, anomalous SSTs preceding the extreme low tide and flood in 1987 that reportedly caused coral bleaching in the central GBR^36^,^37^ may have also lowered the coral’s ability to withstand the later events (Fig. 6a). Therefore it becomes difficult to resolve which of these events (or combination) was primarily responsible for mortality because their timing falls within the limits of the U-Th dating method (±1–2 years). Regardless, the size and frequency (Figs 2e,f and 7) of the larger dead faviids (largest colony sampled was 71 cm in height) suggests that it has been up to 80 years (based on average extension rates for *P. verweyi*) since a disturbance event of similar severity affected this reef.

At Stone Island, no colonies were dated to the event of 1990.4 ± 1.3 (see Supplementary Material for discussion), however, two other periods of mortality were identified and overlap with the timing of mortality of two coral colonies at Bramston Reef; suggesting a broad-scale response to a disturbance (Fig. 6e-g). Although the earlier period of mortality is less well constrained (due to the inherently large ^230^Th age errors), its coincidence with major flooding in 1970 is noteworthy^38^. Similarly, the 2007.2 ± 1.4 period of mortality precisely brackets the timing of extremely large rainfall events that occurred in 2008 where annual Don River discharge exceeded 430,000 ML (Fig. 6c). This event was larger than the flood that took place in 1991, yet only one coral from Bramston Reef dated to this time period which we attribute to the limited recovery of coral communities by this time. While the discontinuous river discharge record for the region makes it difficult to draw any statistically robust correlations, the overlap between the ^230^Th age data and the timing of these events suggests that factors associated with major flooding may be primarily responsible for mortality in coral communities in this region. The lowered salinity from the flood plume could also have been responsible for mortality on the reef slope at both Bramston and Stone Island, resulting in the low level of coral cover observed in 2012 (Supplementary Movie S1 and S2). Large freshwater plumes have been documented to cause 100% mortality on reef flat environments down to depths of 4 m^39^.

Based on the ^230^Th age data, more than 20 years have passed since the events that took place around 1990.4 ± 1.3 AD killed the large faviid colonies depicted in the 1994 images of Bramston Reef. While the limited range of view of the photographs and lack of quantitative data in c.1890 makes it difficult to draw any robust conclusions on whether or not there has been an overall decline in coral cover, the descriptions of Bramston Reef by Saville-Kent^20^ provide an invaluable reference to compare modern coral community composition. By 2012, coral cover was 7.4 ± 6.4% with genera such as *Goniastrea* and corymbose *Acropora* spp. described by Saville-Kent in c.1890 having re-appeared, suggesting that there has been some recovery in coral communities since 1990.4 ± 1.3 AD. Although macroalgal cover was high in 2012 (45 ± 9%), a similarly ‘luxuriant crop of seaweeds…mixed with coral growths, (was) conspicuous…[p. 15 (ref. 20)]’ in c.1890, suggesting that high algal cover was a typical part of the landscape before many of the major modifications to the adjacent catchment area took place (with the exception of dredging in c.1880). However, because this area of the reef was largely dominated by massive faviid colonies estimated to be between 25 and 81 years of age, full recovery of coral communities to pre-flood demographics may take several decades longer.

These observations are in stark contrast to those made at Stone Island. Once characterized by high coral cover in c.1890 (ref. 20) and 1915 (photographer unknown) (Fig. 4a,b), the entire reef flat was wiped out by flooding in 1918 and showed no signs of recovery by 1925 (ref. 34). Hedley^34^ described the reef as though all coral had ‘been planed away by the waves as if some huge razor had shaved off the coral growth down to the low tide level’ (p.37). In 1953 (28 years later), negligible recolonization by hard corals was reported^40^.

While local residents say that there was a healthy reef flat at Stone Island sometime during the 1960–1970s^19^, to what extent coral communities recovered in comparison to its pre-1918 state remains uncertain without any photographic evidence. The three dead *in situ* coral colonies dating to around 1969 ± 8.8 provides some support to suggest that living corals were in fact present around this time. If so, then the estimated time between disturbance and recovery of coral communities at Stone Island may have been between 40–50 years.

Yet by 1994 Stone Island had returned to a state similar to the descriptions made in the earlier part of the Century and appears to have equally limited coral cover until the latest surveys in 2012 (Fig. 4c–f). The age of four dead *in situ* corals (including plating *Acropora* sp. and *Cyphastrea serailia*) precisely dating to 2007.2 ± 1.4 AD suggests that living corals were present until very recently, although coral cover is unlikely to have been high because the time period between 1994 [where no *Acropora* and very few massive coral colonies were found^19^] and the 2007.2 ± 1.4 event (~10–13 years) may not have been sufficiently long enough for communities to recover. It is also possible that colonies may have been transported off the reef by strong winds and waves, resulting in very few *in situ* dead corals available for sampling in 2012. Indeed, photographs and descriptions of the damage at Poole Island [less than 8 km south-east of Stone Island and Bramston Reef (pg. 50 (ref. 20)] and Saddleback Island [~26 km east of Edgecumbe Bay (plate XXXI (ref. 20)] caused by a cyclone that passed within close proximity of Bowen in 1884, suggests that this area is not immune to physical disturbance. Following the period of mortality in 2007.2 ± 1.4, a category three and category two cyclone passed within 100 km of the Bowen region in 2010 and 2011, respectively, which could have removed all traces of the once living community (Fig. 6d; Supplementary Table S5). Nevertheless, the persistence of the dead coral rubble field for the most part of the last century is strong evidence to suggest that Stone Island coral communities have been unable to fully recover to pre-disturbance levels since the major flood event that first wiped them out in 1918.

Previous studies on recovery of coral communities following disturbance on reef flat and reef crest environments have shown that reefs that were fast to recover (1–10 years) either had low levels of mortality and/or obtained high coral cover as a result of the regeneration of surviving tissue e.g.^41^,^42^. Slower rates (>10 years) were observed when coral mortality was high and there were changes to the physical environment, in some instances taking more than 20–30 years to achieve only partial recovery^43^,^44^. Reports of disturbance and recovery at Snapper Island, an inshore fringing reef 5 km from the mainland in the northern GBR, perhaps provides the most reliable comparison with which to compare disturbance dynamics of Bramston Reef and Stone Island: all three are fringing reefs lying within 5 km of the mainland and with a similar disturbance history. In 1996, major flooding of the Daintree River caused almost complete coral mortality on the reef flat and upper slope on the southern side of Snapper Island, resulting in a decline in coral cover from ~88% to less than 15%^39^. Later in 1999, a tropical cyclone turned most of the dead *in situ* acroporids to rubble and overturned many of the large massive coral colonies^45^. Thereafter coral cover remained low (~10%) before gradually increasing to ~18% cover in 2005, 39% cover in 2009 and 50% cover in 2012 (refs 5 and 45). While present day coral cover only represents half that of pre-disturbance levels, it is important to note that there has in fact been an increase in cover over a 13 year time window.

For Bramston Reef, there has been some increase in coral cover following the 1991 ± 1.3 AD disturbance event over a ~21 year time period. For Stone Island, the limited evidence of coral growth since the early 19th Century suggests that recovery is severely lagging. Even if a ‘healthy’ community did exist in the 1960–70s, it still took 40–50 years to recover following the 1918 disturbance event. These timescales are similar to (or in the case of Stone Island exceed) documented recovery rates of reefs that have undergone almost 100% mortality, have experienced changes to the reef structure or reflect a chronically disturbed (e.g. eutrophic) environment (Supplementary Table S6).

While the visual impact of the previously healthy versus currently dead reef in the photographs is attractive in its use as evidence for a decline in inshore reef health on the GBR [e.g. GBR Outlook report^46^], caution is warranted in attributing this delayed recovery to anthropogenic influences without first considering long-term geomorphological processes^21^. Stratigraphic evidence from reef matrix cores suggests that most inshore and fringing reef development occurred between ~8,000 and 5,000 yBP under naturally turbid conditions^47^. Indeed, radiocarbon (^14^C) ages obtained from fossil microatolls sampled across the reef flat at Stone Island revealed much of the development to have taken place between 6,870 ± 280 and 6,090 ± 190 yBP (recalibrated ages by^48^ based on^26^). The limited seaward progradation or ‘slowing down’ in reef growth from the late Holocene to present has been attributed to a lack of available substrate, stabilization and subsequent fall in sea level during the post marine transgression (at 5,500–4,800 yBP) and an accumulation of the inshore sediment wedge that is likely to have led to a rise in ambient turbidity^47^. As a result, many fringing and nearshore reefs have naturally senesced, and where corals are present they may represent communities growing at their environmental extremes^21^. However, there are a many examples of turbid reef crest environments similar to those investigated in this study that can represent highly diverse coral reef habitats^45^,^49^,^50^ with actively accreting reef slopes^51^. Although elevation surveys performed at Stone Island reveal limited accommodation space for reef growth on the shallow reef crest and back reef zone, there remains ample substrate below the MLWS limit for coral growth on the extensive reef flat and reef slope (Fig. 3b); yet coral cover is extremely low (0.09 ± 0.12%; Fig. 5). This is in contrast to Bramston Reef, where living corals contributing to 7.4 ± 6.4% hard coral cover can be found at similar elevations (Fig. 3a). Thus, while the proximity of the reef substrate to MLWS height could explain the apparently degraded state of the reef crest and back reef environments at Stone Island, it does not explain the low level of coral cover observed across the entire reef.

Other possible reasons for the negligible recolonization of corals at Stone Island include a lack of structural complexity that is essential in creating habitat for newly settled recruits to survive^43^,^52^. Unlike the hummocky relief of Bramston Reef (Fig. 2), the reef substrate at Stone Island appears flat and infilled with very little three-dimensional structure (Fig. 4). In a recent study by Graham *et al.*^53^, factors such as water depth and structural complexity were found to be key predictors of ecosystem trajectories following major disturbance events, with shallow water reefs (<6.6 m depth) characterized by low structural complexity undergoing regime shifts from coral to macroalgal dominated. The large amount of unconsolidated rubble is also likely to lead to high levels of mortality when the young corals attached to the loose rubble framework are rolled by waves, collapse or crumble^54^,^55^,^56^. Hydrological changes could be another contributing factor, as recent studies have discovered two small gyres in the northern and southern end of Edgecumbe Bay that are believed to cause very slow flushing of coastal waters; indeed almost stagnant waters (Wolanski unpublished data in^57^) which may prevent coral larvae from reaching Stone Island. A chronic decline in water quality as a result of a long history of catchment modification within Edgecumbe Bay (see Supplementary Material) may also be affecting the overall health and survival of corals in the area. However, there is a deficiency in estuarine and offshore monitoring that makes it difficult to assess any downstream impacts from the high concentrations of herbicides, pesticides and nutrients measured upstream^58^. All of these factors require further investigation, in addition to ongoing monitoring of coral communities, which will be necessary to follow their progress (or lack thereof) towards recovery.

Overall, this study provides robust baseline information on the timing and potential cause/s of the changes observed in a collection of historical photographs, as well as a reference point for ongoing monitoring. The combination of anecdotal historical evidence (photographs) together with quantitative geochemical and ecological data is a unique and valuable method to reef managers to fill the knowledge gap that exists prior to monitoring programs. A similar approach can be applied to other reef locations where an accurate chronology for the timing of substantial declines in coral cover, shifts in species composition and associated drivers are required.

## Methods

### Study sites

**Figure 1:**
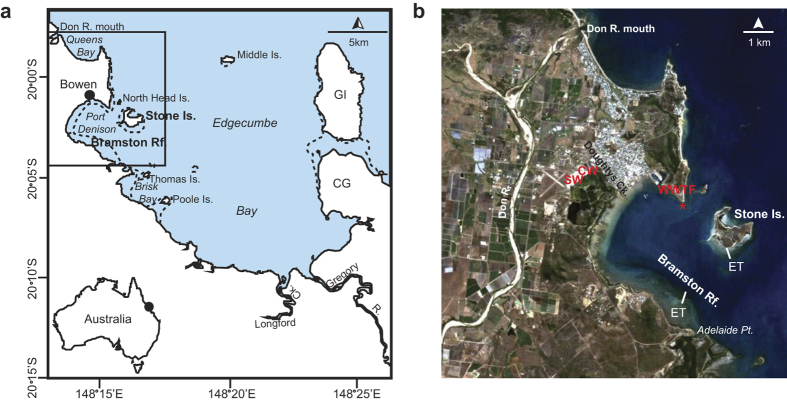
Sampling location. (**a**) Map of Edgecumbe Bay. Major rivers influencing this area include the Don and Gregory Rivers, as well as several other streams draining directly into the bay. Gloucester Island (GI) and Cape Gloucester (CG) are also labelled as they appear as major landmark features in the background of the historical photographs (Figs 2 and 4). Bramston Reef and Stone Island are located at the northern end of the bay. Map was created using Adobe Illustrator CS6 software; (**b**) Satellite image of Bowen region taken in 2009 (Source: Geoscience Australia. Licensed CC) showing the location of Bramston Reef and Stone Island and respective elevation transects (ET). This image clearly shows the extensive land use in the area. Major industries include the Bowen Coke Works (CW), Saltworks (SW), waste water treatment facility (WWTF) and approximate location of discharge outlet (*) (See Supplementary Information).

Bramston Reef (20°03′S; 148°15′E) and Stone Island reef (20°02′S; 148°17′E) are a nearshore and fringing reef, respectively, that are located within Edgecumbe Bay, central GBR (Fig. 1). Neighboring catchment areas include the Burdekin, Don and Proserpine Basin (Mackay-Whitsunday region), where land use is predominantly grazing and sugarcane production^31^. The land adjacent to Edgecumbe Bay also has a long history of industrial activity and coastal development since the establishment of the Bowen Township in 1861 AD (Fig. 1b, Supplementary Information). Runoff to this area is primarily received from the Gregory River at the southern end of the embayment, occasionally the Don River to the north^59^ as well as several other smaller streams.

**Figure 2:**
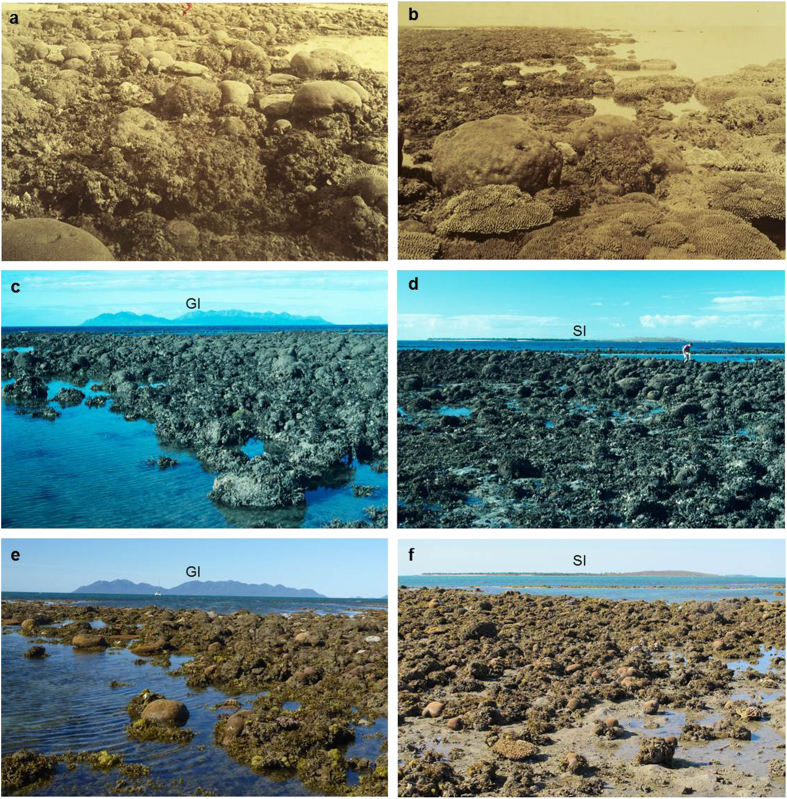
Historical and modern photographs of Bramston Reef. (**a,b**) Historical photographs of Bramston Reef taken by William Saville-Kent^20^ in c.1890. In the photograph on the left, Saville-Kent described the large conspicuous faviids as being ‘thickly crowded’ with ‘ovate symmetry and peculiar incidence…(that) if transported to an ordinary landscape, they would pass for a flock of sheep.’ (p.15) Photographs of Bramston Reef taken in 1994 (**c**) looking south-east towards Gloucester Island (GI) and (**d**) looking north-east towards Stone Island (SI) [Photographer: A. Elliot (© Commonwealth of Australia GBRMPA)]. (**e,f**) Modern photographs taken during this study in 2012 at the same locations as the photographs taken in 1994 (Photographers: H. Markham (left) and N. Leonard (right)).

**Figure 3:**
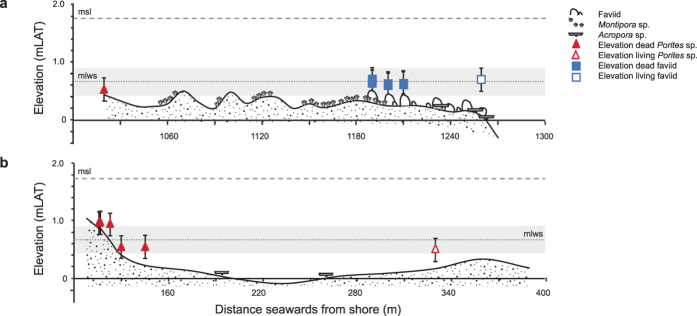
Bramston Reef and Stone Island elevation profiles. Elevation transects were (**a**) 250** **m long) (starting ~1,020** **m from shore) for Bramston Reef, and (**b**) 280** **m long (starting ~110** **m from shore) for Stone Island, running perpendicular from the shore to the location of the historical photographs at the reef crest. Note that most of the reef flat and reef crest for both reefs is below mean low water spring (MLWS) height (0.67 mLAT) which is considered the upper limit of open water coral growth^21^,^22^ (with the exception of the start of the elevation transect at Stone Island). Filled and unfilled squares represent the elevation of living and dead faviid colonies relative to LAT (±20%), respectively. Filled and unfilled triangles represent the elevation of living and dead *Porites* microatoll colonies relative to LAT (±20%), respectively.

In c.1890, images of Bramston Reef taken in the vicinity of Adelaide Point by Saville-Kent^20^ depict large living tabular *Acropora* spp., massive faviid and *Porites* corals (Fig. 2a,b; Supplementary Fig. S1). In 1994, Wachenfeld^19^ revisited Bramston Reef in an attempt to assess how these coral communities have changed over time. The same area photographed in c.1890 was located by identifying the characteristically large faviid colonies depicted in the earlier images during mean low water spring (MLWS) tides. However, because there is no geographical feature in the background to be able to determine the exact site of the c.1890 historical photographs, the modern photographs taken in 1994 must be considered as representative photographs of the same reef flat, not exact replicas^19^. Nevertheless, the older photographs can still be used to make valid comparisons between the type of coral communities existing in the area at different periods in time. In 1994, the majority of the large faviid colonies were found to be dead (with the exception of smaller colonies <15 cm diameter) and no large living tabular *Acropora* spp. were present at the site where the photographs were taken (Fig. 2c,d; Supplementary Fig. S2).

**Figure 4 f4:**
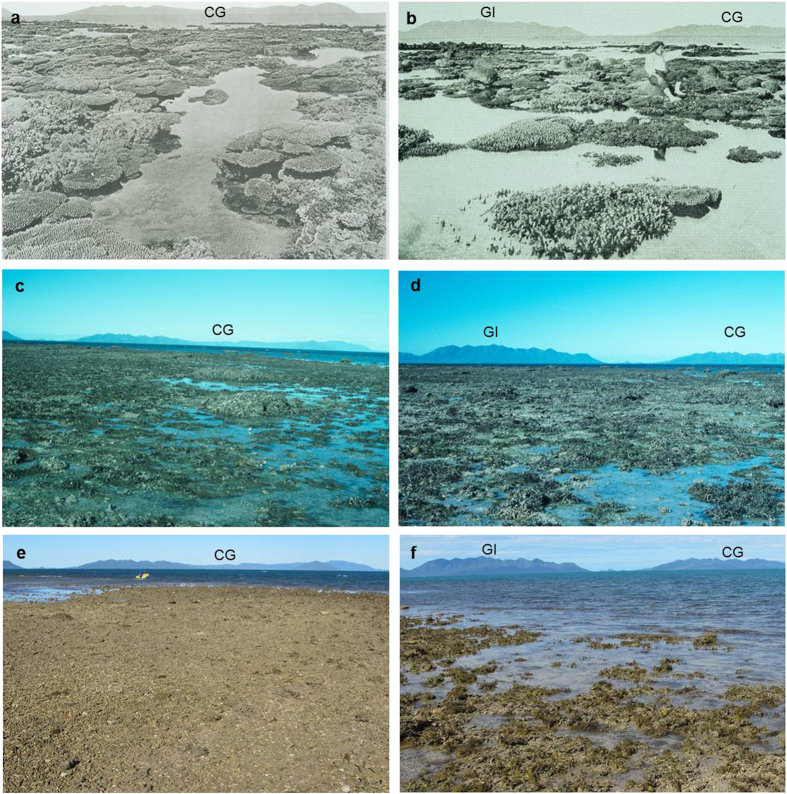
Historical and modern photographs of Stone Island. (**a**) Historical photograph taken by William Saville-Kent^20^ in c.1890 depicting high cover of branching and tabular *Acropora*; (**b**) Historical photograph taken in 1915 showing similarly high coral cover with large faviid colonies in the background of the image (photographer unknown); (**c,d**) Photographs of Stone Island taken in 1994 [Photographer: A. Elliot (© Commonwealth of Australia GBRMPA)]; (**e**) Modern photograph taken during this study on 30 July 2012 representing a time-series for images a and c (Photographer: T. Clark); (**f**) Modern photograph taken during this study on 31 July 2012 representing a time-series for images (**b**) and (**d**) (Photographer: H. Markham). Geological features in the background of the images used to identify the location of the historical photographs include Gloucester Island (GI) and Cape Gloucester (CG).

**Figure 5:**
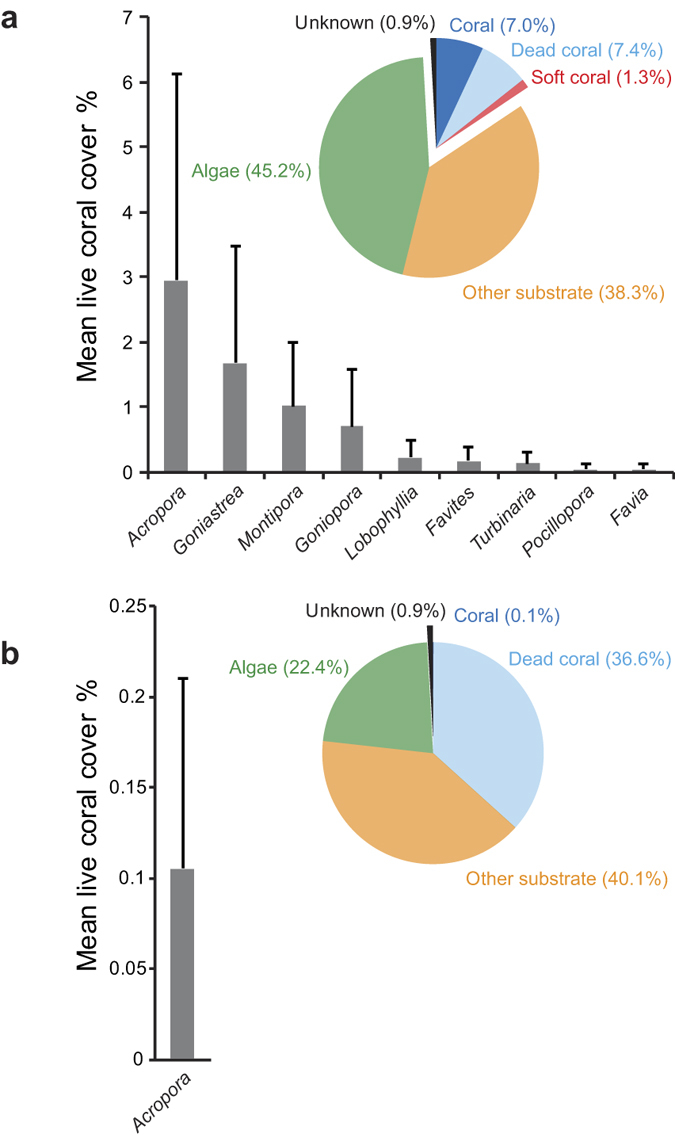
Modern benthic structure in 2012. Mean percent coral cover (±1 sd) for the recorded genera and pie graph of percent cover of major benthic categories [including live coral, dead coral, soft coral, algae, other substrate (including sand, sediment) and unknown] for (**a**) Bramston Reef, and (**b**) Stone Island.

**Figure 6:**
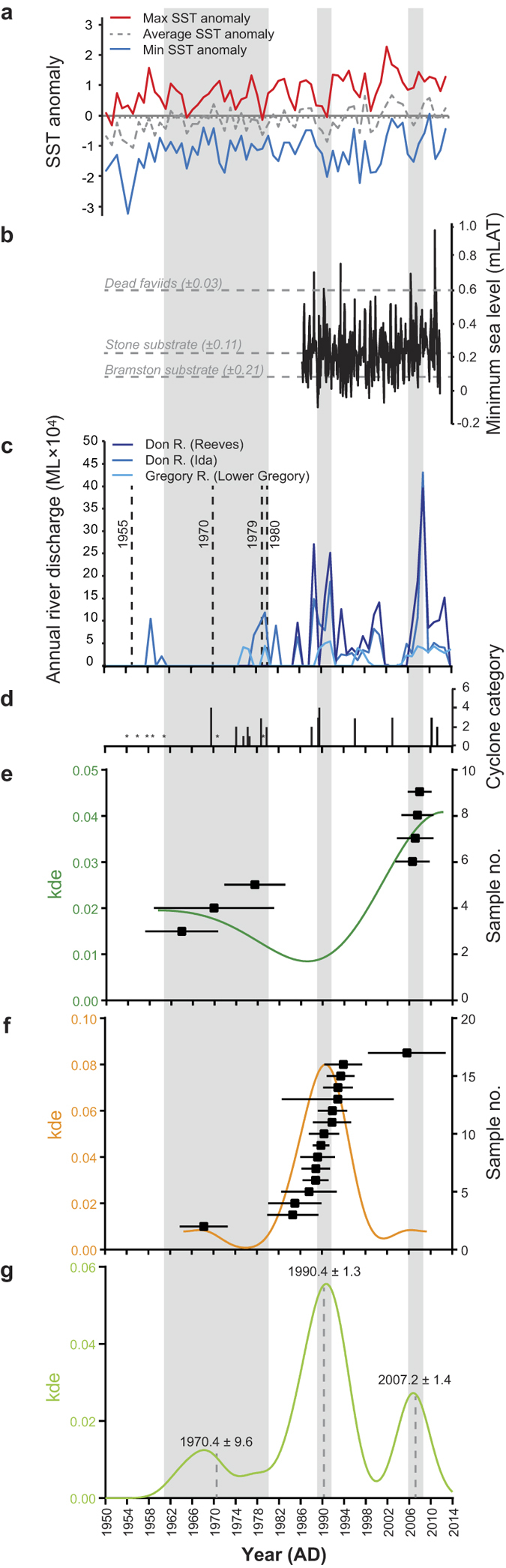
Timing of mortality for the dead *in situ* corals sampled at Bramston Reef and Stone Island compared with environmental records. (**a**) Annual maximum, average and minimum sea surface temperature (SST) anomalies (HaddSST2) for 5 × 5 grid 147.5 °E, 17.5 °S from 1950 to 2014 (Source: Hadley Centre); (**b**) Monthly minimum tidal gauge data (mLAT) for Bowen station number 061007A from 1986 to 2013 (Source: Queensland Environmental Protection Agency). Average elevation of the dead faviids, Bramston and Stone Island substrate is also shown (± 1sd); (**c**) Annual river discharge for the Don River at Reeves (station number 121003A), Don River at Ida (station number 121001A) and Gregory River at Lower Gregory (station number 122004A)(Source: Queensland Department of the Environment and Resource Management). Vertical dashed lines denote known flood event with no available discharge data^38^. Note that there was an extremely large discharge event of 285,601 ML for the week beginning 4/02/1991 associated with intense rainfall following cyclone Joy; (**d**) Cyclone events passing within 100** **km of Bowen (Source: Australian Bureau of Meteorology). *denotes known cyclone event with no available data on cyclone category (Supplementary Table S5); (**e**) Kernel density estimate (KDE) distribution for seven U-Th ages obtained from dead *in situ* corals sampled at Stone Island (smooth curve). The height and width of the KDE curve reflects a function that stacks a Gaussian ‘bell curve’ on top of each measurement and whose standard deviation is determined by the local probability density^63^; (**f**) KDE distribution for 16 U-Th ages obtained from dead faviid colonies collected at Bramston Reef (smooth curve). Sample BRS1T2.1 (1853.0 ± 2.8 AD) falls outside the range of the x-axis; (**g**) KDE distribution for all 23 U-Th ages obtained from both Stone Island and Bramston Reef. Each peak in the distribution is centred around a weighted mean age of 1970.4 ± 9.6, 1990.4 ± 1.3 and 2007.2 ± 1.4 AD.

**Figure 7:**
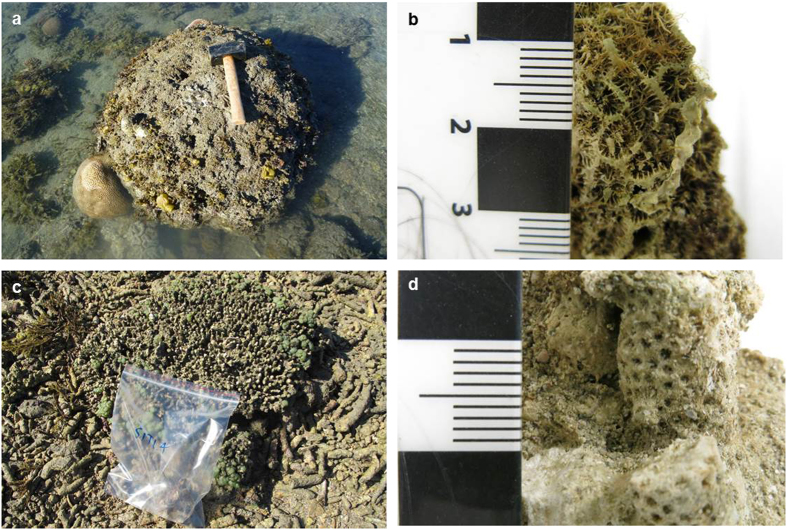
Example of *in situ* dead coral colonies sampled in this study. (**a**) Faviid colony prior to sampling at Bramston Reef. The hammer in the photograph is 26** **cm in length; (**b**) Excellent preservation of corallite structure on many of the dead colony surfaces suggests minimal erosion; (**c**) Tabular *Acropora* sp. colony prior to sampling at Stone Island (width of sample bag in the photograph is 23** **cm) and (**d**) Subsample taken from the same colony for U-Th dating (Photographer: T. Clark).

The images and descriptions of the reef at Stone Island taken in c.1890 reveal a reefscape dominated by branching *Acropora* alongside other coral genera including *Montipora*, *Goniastrea*, *Fungia* and *Turbinaria*^20^ (Fig. 4a). While a similar reef was observed in 1915 (Fig. 4b), by 1994 the reef was covered in a mixture of coral rubble and algae with no living *Acropora* and very few massive coral colonies present during spring low tide^19^.

### Modern photographs and benthic surveys

Using the geographical features in the background of the photos taken in 1994 as a reference 19, a similar photograph was retaken and the GPS coordinates recorded at Stone Island (30 July 2012) and Bramston Reef (31 July 2012). To quantify modern (living) coral community composition and provide a baseline reference for future comparison, four 20 m transects were laid parallel to the reef crest at Bramston Reef and photographed at 1m^2^ intervals at low tide using a Canon G12 camera and later analysed with Coral Point Count with Excel extensions^60^ using 30 points per quadrat. Only two 20 m transects were used at Stone Island as the reef crest environment was highly consistent. Community structure was categorized by quantifying total cover of: live hard coral, dead coral, soft coral, algae, other substratum (substrate and sediment), and unknown. Other organisms, such as anemones, large molluscs, hydroids and ascidians, were rarely encountered and not included in the analysis. Hard coral cover was further categorized into dominant genera typically found in near-shore environments (such as *Acropora, Goniastrea, Montipora, Favia, Goniopora* and others).

### Elevation surveys

For the Bowen region, mean sea level (MSL) and MLWS height is 1.76 mLAT and 0.67 mLAT, respectively^61^. Using a Magnum-Proshot 4.7 Laser Level and Apache Lightning 2 receiver, substrate/coral colony height was measured approximately every 20 m starting at a distance of ~100 m, and ~1,000 m from shore (due to the limited range of the receiver) to the water edge for Stone Island and Bramston Reef, respectively, (Supplementary Table S1) and referenced to still tide time at three different time points. The mean of the three points was used to reduce measurements to mLAT using tide data from Bowen Storm Surge gauge 061007A, made available by Maritime Safety Queensland (MSQ), with a resultant standard deviation of 0.05 m. However, due to the known limitations of referencing off still tide time we assigned a more conservative error of ±0.2 m. Photographs were also taken of the benthos where each elevation measurement was taken to investigate reef flat zonation.

### Sampling and U-Th analytical techniques

Dead *in situ* coral samples were collected from Bramston Reef and Stone Island in order to constrain the timing of coral loss observed in the historical photographs. To determine the time of death of the massive *in situ* faviids present in the 1994 photographs of Bramston Reef, a subsample of carbonate material was collected from the top of 17 randomly selected colonies using a hammer and chisel along transects 1, 2 and 4 (the rising tide did not permit us to sample along transect 3), as well as along the elevation transect. Care was taken to avoid sampling bias with both small and large colony sizes sampled (range 22–71 cm). Given that the colonies were covered in mud and algae at the time of collection, this also prevented any bias towards preservation state. Only nine dead corals believed to be *in situ* (upright growth position, attached to the benthos or with minimal evidence of breakage) were found along transects 1 and 2 at the reef crest of Stone Island.

Samples were identified to species level in the laboratory and high-purity (free from any visible signs of contamination or alteration) aragonite material from each of the dead faviid colonies was carefully vetted under a light microscope then prepared for U-Th age dating on a Nu Plasma Multi-Collector Inductively Coupled Plasma Mass Spectrometer (MC-ICP-MS) in the Radiogenic Isotope Facility at the University of Queensland following methods and procedures described in Clark *et al.*^24^,^25^. Following measurement, all U-Th ages were calculated with the Isoplot/Ex 3.00 program^62^ (Supplementary Table S4).

### Initial ^230^Th correction

All U-Th ages were corrected for initial/detrital ^230^Th contributions in order to obtain precise and accurate ^230^Th ages for such young coral samples. The detrital ^230^Th end-member for corals sampled from Bramston Reef was determined from a ^230^Th/^232^Th versus ^234^U/^238^U isochron by analysing three samples of coeval material from each of the following colonies: BRS1-R2 (BRS1-R2a, BRS1-R2b, BRS1-R2d) and BRS1T2.3 (BRS1T2.3a, BRS1T2.3b, BRS1T2.3c; Supplementary Fig. S4). Approximately 1 g of sample material was collected from each colony, divided into three parts and each subsample exposed to varying degrees of pre-cleaning (uncleaned, ultrasonicated in Milli-Q or soaked and sonicated in H_2_O_2_ and visually inspected under the microscope). U-Th data for each sample was plotted in ^230^Th/^232^Th versus ^234^U/^238^U space and the ^230^Th/^232^Th_0_ ratios calculated from the y-intercept (with 2σ). The detrital component for the site was determined to be 0.81 ± 0.01 (1σ), based on the average of the ^230^Th/^232^Th_0_ ratios. This value, together with a GBR weighted mean ^230^Th/^232^Th_0_ value of 1.066 ± 20% determined by Clark *et al.*^63^, was used in a two-component mixing equation to correct for detrital and hydrogenous ^230^Th present in the dead coral samples and allow for accurate ^230^Th age estimates^24^.

### Surface age determination

**Figure 8:**
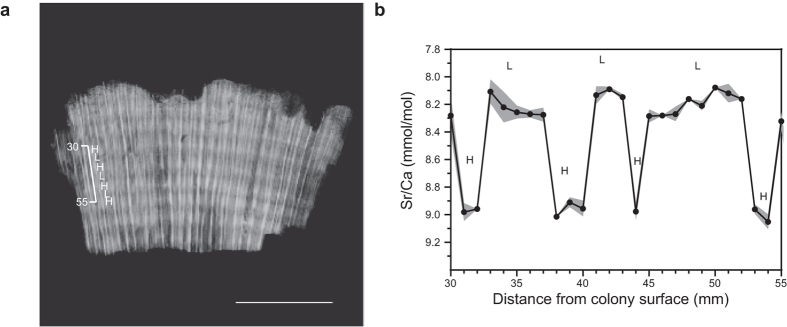
Growth characteristics of *P. verweyi* sampled at Bramston Reef. (**a**) Example of annual density banding patterns revealed by positive X-radiographs. Mean annual linear extension for high and low density banding couplets was determined to be 0.99 ± 0.16 (1sd) cm yr^−1^. Scale bar is 5** **cm in length. (**b**) Elemental Sr/Ca ratio data (±1sd) representing ~3 years of growth obtained for the same *P. verweyi* coral sample. Each high and low density band was found to correspond to high and low Sr/Ca values, respectively, reflecting seasonal changes in sea surface temperature [see also Weber *et al.*^65^]. Thus, each low and high density-banding couplet corresponds to one year of growth.

The mortality age (or surface age) of each sampled colony was determined by subtracting the number of annual growth bands above the sampling location (deduced from coral X-rays) from the corrected ^230^Th age (years)^25^. For samples where density banding was unclear, the number of years subtracted from the ^230^Th age was calculated by dividing the distance from the surface of the colony to the sampling location (cm) by the average linear extension rate (cm yr-1) for that coral species. Average linear extension rates for P. verweyi colonies and G. aspera colonies were determined to be 0.88 ± 0.23 (± 1sd) cm yr^−1^ and 0.73 ± 0.05 cm yr^−1^, respectively, based on dead coral colonies that were slabbed into a 7 mm thick section down the main growth axis and X-rayed (100 mA, 0.025 sec, 50 kV, 120 cm) to be able to measure the length of each annual density banding couplet (Fig. 8a; Supplementary Table S2). The high and low density band couplets were further verified as one year of growth by measuring Sr/Ca ratios at less than monthly resolution (four to ten samples per year) due to the brittle nature of the coral skeleton (Fig. 8b; Supplementary Information). Kernel Density Estimator (KDE) values^64^ derived from the surface ages were then created using the statistical program R version 3.0.2 (R Core Team 2013) to produce a ^230^Th age distribution for comparison with long term instrumental or proxy environmental time-series available for the region (including sea surface temperature anomalies, tidal height, cyclone history and river discharge) in an attempt to identify the primary driver for mortality.

## Additional Information

**How to cite this article**: Clark, T. R. *et al.* Historical photographs revisited: A case study for dating and characterizing recent loss of coral cover on the inshore Great Barrier Reef. *Sci. Rep.* **6**, 19285; doi: 10.1038/srep19285 (2016).

## Supplementary Material

Supplementary Information

Supplementary Movie S1

Supplementary Movie S2
