# Supplementary material for: Historical photographs revisited: A case study for dating and characterizing recent loss of coral cover on the inshore Great Barrier Reef
Source: Sci Rep. 2016 Jan 27;6:19285. doi: 10.1038/srep19285 (PMC4728430; doi:10.1038/srep19285)
Supplement: Supplementary Information [file srep19285-s1.pdf]

Historical photographs revisited: A case study for dating and characterizing recent loss of coral cover on the inshore Great Barrier Reef

Tara R. Clark[1*], Nicole D. Leonard[1], Jian-xin Zhao[1*], Jon Brodie[2], Laurence J. McCook[3], David R. Wachenfeld[4], Ai Duc Nguyen[1], Hannah L. Markham[5], John M. Pandolfi[5]

[1]*School of Earth Sciences, The University of Queensland, Brisbane QLD 4072 Australia*

[2]*Centre for Tropical Water and Aquatic Ecosystem Research, James Cook University, Townsville, QLD 4811 Australia.*

[3]*ARC Centre of Excellence in Coral Reef Studies, James Cook University, Townsville, QLD 4811 Australia.*

[4]*Great Barrier Reef Marine Park Authority, 2-68 Flinders Street, PO Box 1379, Townsville, QLD 4810 Australia.*

[5]*ARC Centre of Excellence in Coral Reef Studies, School of Biological Sciences, The University of Queensland, Brisbane QLD 4072 Australia.*

*Correspondence: Email: t.clark1@uq.edu.au; j.zhao@uq.edu.au

Supplementary Information

Absence of ^{230}Th ages dating to 1990.4 ± 1.3 event at Stone Island

One possible reason for the absence of coral colonies at Stone Island dating to the 1990.4 ± 1.3 event that caused extensive mortality at Bramston Reef, is that there were very few living coral colonies present at that point in time. As discussed in the main text, there is anecdotal evidence to suggest that there was a healthy reef flat at Stone Island sometime during the 1960-1970s[1]. Following the destruction of the coral community in 1918, the time between disturbance and recovery is therefore estimated to be between 40-50 years. If there was a mortality event around 1970.4 ± 9.6 (determined by the ^{230}Th ages obtained from Stone Island and neighbouring Bramston Reef), then Stone Island may have still been in the very early stages of recovery with low coral cover; subsequently reducing the probability of being able to date a coral colony to this time period.

A second theory is that remnants of an existing population could have been dislodged from the unstable substrate characterised by loose coral rubble during one of the many category 3 or 4 cyclones that took place in the years leading up to the time of the observations made by Wachenfeld[1] (Supplementary Table S5). On the other hand, the large massive faviid colonies characteristic of Bramston Reef, which is slightly sheltered by Stone Island, remained unaffected by strong waves

Third, the disturbances that occurred in 20th Century resulted in drastic modification of the reef flat and a loss of structural complexity, thus hindering coral re-establishment. Evidence of this comes from Hedley[2], who described the reef as though all coral had 'been planed away by the waves as if some huge razor had shaved off the coral growth down to the low tide level'.

Catchment modification within Edgecumbe Bay

Soon after settlement in 1861, dredging of Port Denison began (c.1880) to maintain accessibility for shipping and was likely to have resulted in the local re-suspension of fine sediments. Other major industries followed, including the Bowen Saltworks (est.1925) and Cokeworks (est.1933), where little is known about what waste products were, and still are, being discharged directly into coastal waters[3]. Other point-sources of pollutants include several large aquaculture farms and the Bowen wastewater treatment facility (est. late 1950s), with the latter only upgraded to secondary treatment in 1989. In 2010, the wastewater facility did not comply with the Department of the Environment's total nitrogen and total phosphorus limits and only 20% of the effluent was being recycled each day. The remaining four megalitres of effluent that was not recycled was being discharged directly into northern Edgecumbe Bay from Dalrymple Point[4] (Fig. 1b).

Grazing, horticulture and sugar cane farming are also major contributors of diffuse sources of pollutants including sediments, nutrients, herbicides and insecticides that enter waterways in runoff during high rainfall events. The effects of these pollutants on corals and coral reef environments are well documented[5-7]. Yet despite the various threats to marine environments within this area, water quality data for the region is either limited or results are not available to the public[3,8]. This deficiency in estuarine and offshore monitoring makes it difficult to assess any downstream impacts[8].

From the upstream data that is available for several rivers and creeks, levels of atrazine and the herbicide diuron exceed trigger values developed by the National Water Quality Management Strategy (NWQMS) [3]. Surveys of the Gregory River in 2000-2002 also revealed total nitrogen, total phosphorus and total suspended solids to exceed trigger values[8,9]. The amount of sediment being delivered to the region from the neighboring Mackay-Whitsunday and Burdekin catchments is estimated to have increased 6.0 and 7.8 fold to 1.5 and 4.7 million tonnes compared to pre-European conditions, respectively, of which ~87% can be attributed to human activities[10]. Other tell-tale signs of the effects of poor water quality come from recent reports of increased marine turtle stranding events attributable to the high loading of heavy metals and toxicants in Edgecumbe Bay[3].

Elemental ratio analysis

Methods

The high and low density band couplets were further verified as one year of growth by measuring Sr/Ca, Mg/Ca and U/Ca ratios at approximately monthly resolution in the *P. verweyi* sample Bram_R8. This sample was chosen for analysis as enough material was available to represent several years of growth. Moreover, the X-ray images revealed clear density banding patterns. Approximately 0.001 g of skeletal material was carefully milled at ~1 mm increments (approximately monthly resolution) along the coral's main growth axis using a hand held drill and diamond drill bits. Powdered material was collected on weighing paper and each drill bit replaced between samples to prevent cross-contamination.

A stock solution of each sample was prepared by dissolving ~0.0002 g of powdered material in 12 ml of 2% HNO3 in a pre-cleaned HDPE tube to achieve a dilution factor of 60,000 times. The solution was then shaken to ensure complete homogenisation and centrifuged at 4,000 rpm for 10 minutes.
JCP-1 coral standard and an in-house coral standard were used as external monitors to correct for interference and prepared by dissolving powdered material in 2% HNO3 to achieve a final dilution factor of 60,000 times. An internal standard solution containing known concentrations of Sc, V, ^{206}Bi, ^{235}U was prepared by diluting each element to 60 ppb in 2% HNO_3. The 2% HNO3 solution used to dissolve the samples was used as a blank.

Samples were then measured fully automatically, with the external monitors, internal standard and blank solution introduced separately to an Agilent 7900 inductively coupled plasma mass-spectrometer (ICP-MS) at the Radiogenic Isotope Facility, The University of Queensland. Samples were measured twice to assess reproducibility among samples. Samples were corrected for blank contamination and internal drift using the 2% HNO_3 blank solution and internal standard solution respectively. Elemental concentrations for Sr, Ca, and Mg were calculated using the software MassHunter 2015. Elemental ratios for Sr/Ca, Mg/Ca, U/Ca and Ba/Ca were converted to mmol mol^{-1} using JCP-1certified values

Results

Sr/Ca ratios display a clear cyclical pattern with values ranging from 8.077 ± 0.011 (1sd) to 9.051 ± 0.054 mmol mol^{-1}. This is a slightly lower range compared to values reported in massive *Porites* corals elsewhere in the GBR (ranging ~8.5 to 9.6 mmol mol^{-1}) that are routinely used for climate and environmental reconstruction[11-13]. The time series represented by the Sr/Ca data spans approximately three complete cycles (trough to trough), with each cycle representing one year of growth. High Sr/Ca ratios were found to correspond to high density bands, while low Sr/Ca ratios corresponded with low density bands. Mg/Ca ratios ranged from 3.6051 ± 0.0058 to 5.467 ± 0.058, also producing three cycles between high and low values. Mg/Ca data also followed a similar pattern to U/Ca ratios which ranged from 0.9999 ± 0.0019 to 1.2 ± 0.01 and also producing three cycles. Reproducibility of Sr/Ca for the in-house external monitor and JCP-1 was 0.44 (N=9) and 0.49% (N=9), respectively. Reproducibility of Mg/Ca for the in-house external monitor and JCP-1 was 0.51 (N=9) and 0.38% (N=9), respectively, and 1.23 (N=9) and 0.96% (N=9) for U/Ca.

Supplementary Figures

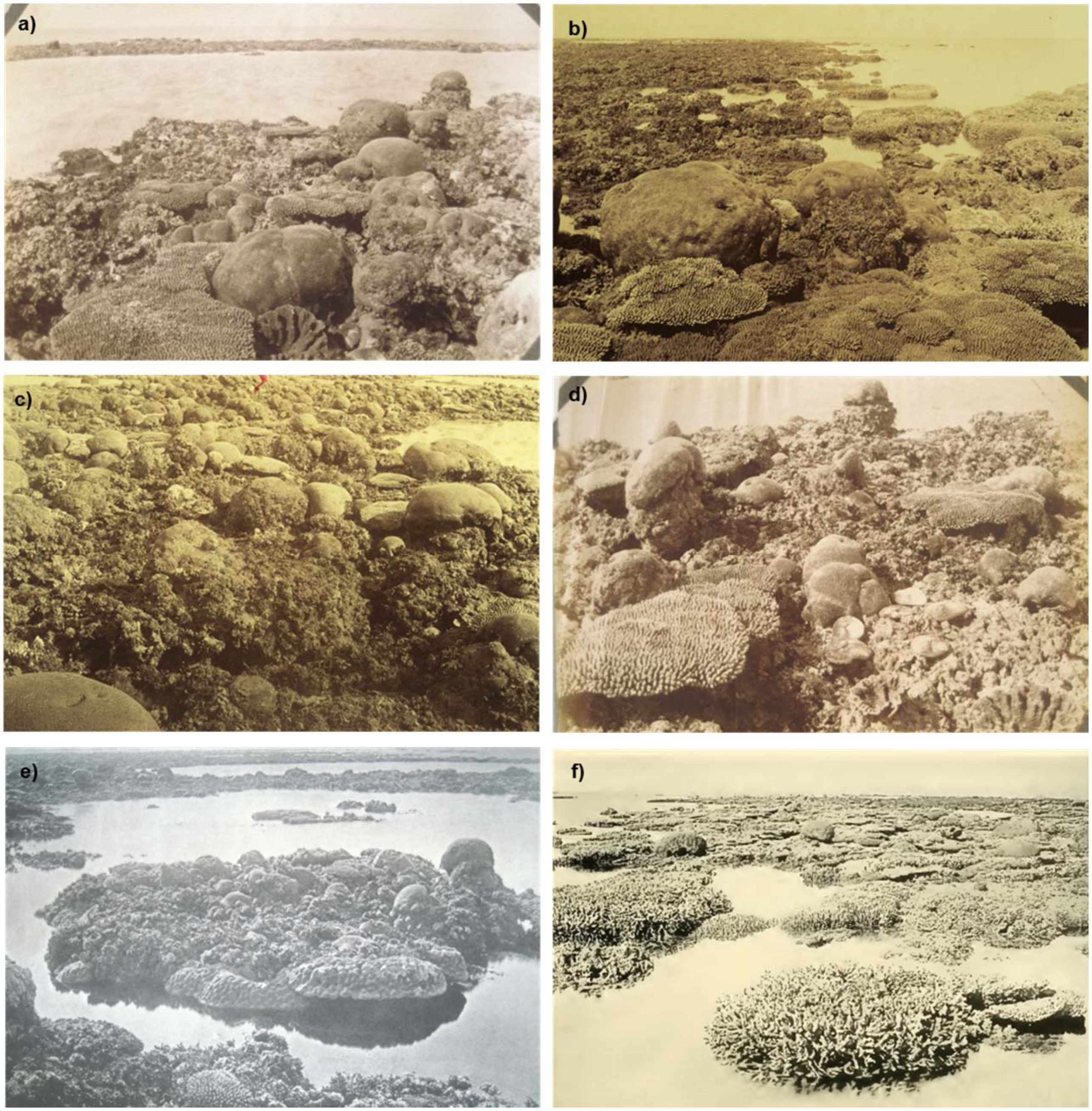

Supplementary Figure S1 | Historical photographs of Bramston Reef. Photographs taken by William Saville-Kent[14] in c.1890. While a geographical landmark is absent in these photographs, comparisons between the type of coral communities existing in the area can still be made with modern photographs to assess any changes in the presence or absence of coral genera.

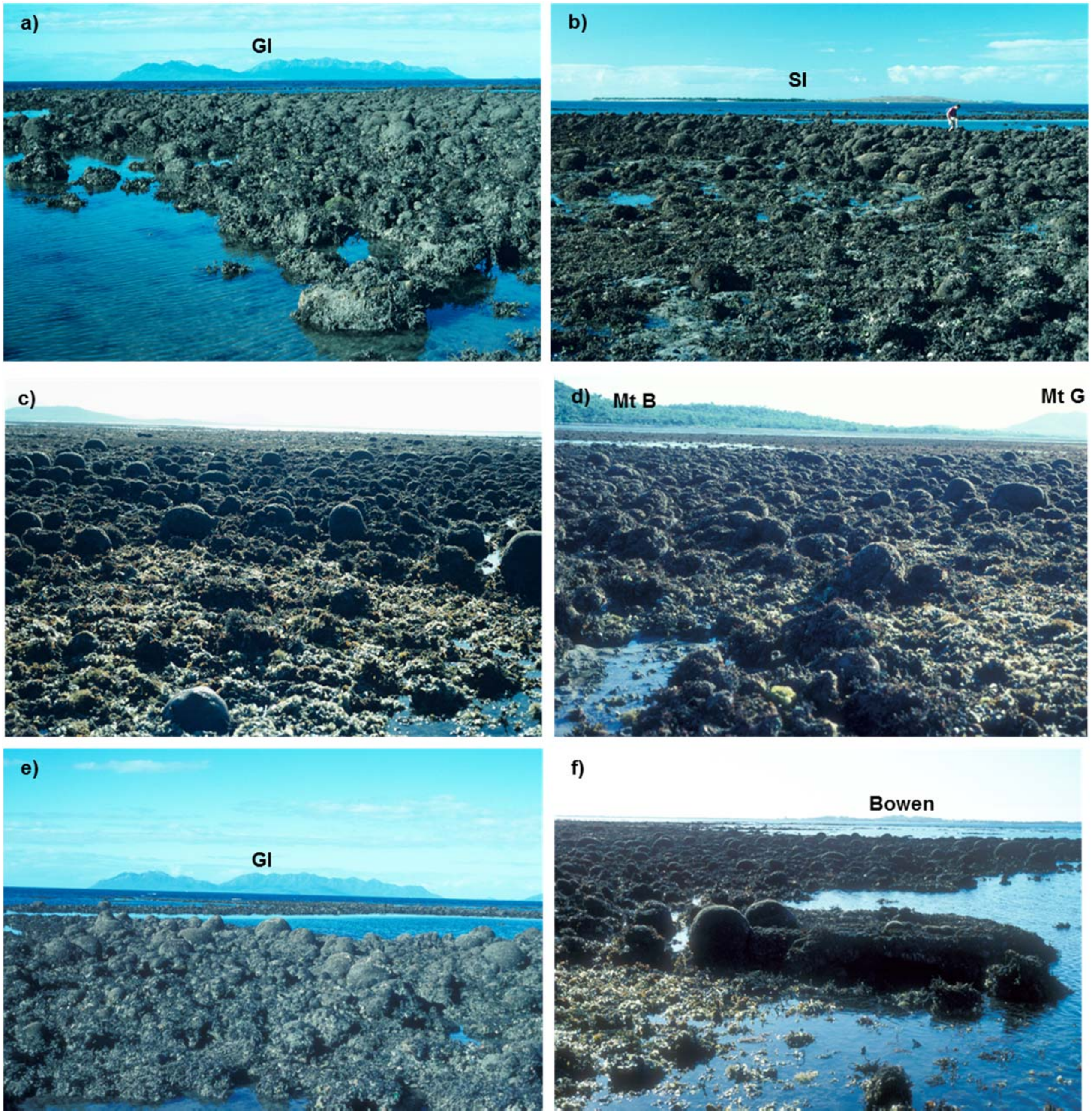

Supplementary Figure S2 | Photographs of Bramston Reef taken in 1994. (a) Looking south-east towards Gloucester Island (GI); (b) Looking north-east towards Stone Island (SI); (c-d) Looking north-west towards the mainland with Mt B and Mt G in the background; (e) Further back from the reef crest looking south-east towards GI; (f) Looking north towards the township of Bowen. Note the large microatoll in the foreground. Photographer: A. Elliot (© Commonwealth of Australia GBRMPA).

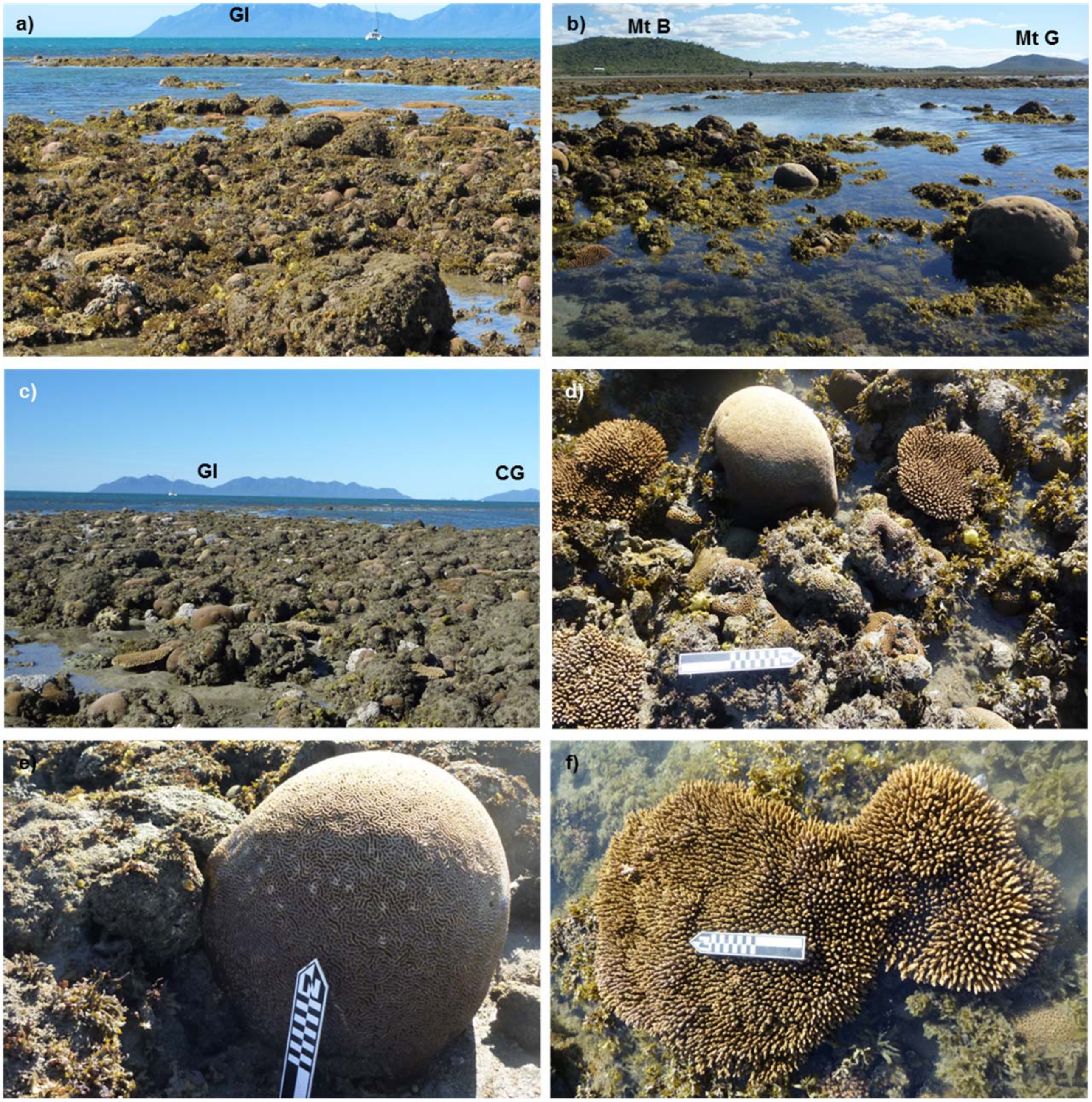

Supplementary Figure S3 | Living corals found at Bramston Reef in 2012. (a) Looking south-east towards Gloucester Island (GI); (b) Looking north-west towards Mt Bramston (Mt B) and Mt Gordon (Mt G); (c) Looking towards GI and Cape Gloucester (CG); (d) Living coral colonies (tabular *Acropora* sp. and faviids) found close to the reef crest; (e) large living faviid colony; (f) large living tabular *Acropora* sp. (Photographer: N. Leonard).

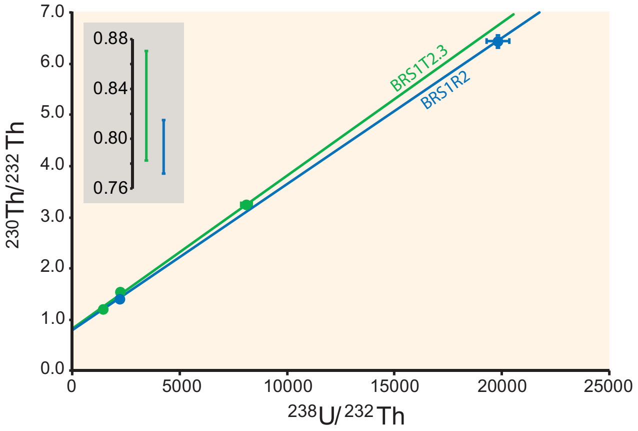

Supplementary Figure S4 | Isochron for constraining detrital ^{230}Th/^{232}Th end-member. A ^{230}Th/^{232}Th versus ^{234}U/^{238}U isochron for two coeval sets of sub-samples obtained from dead faviid skeletons collected from Bramston Reef. Inset shows the isochron-inferred ^{230}Th/^{232}Th$_0$ ratios (y-intercepts with 2σ errors) of the detrital component [average 0.81 ± 0.02 (1σ)].

Supplementary Tables

Supplementary Table S1. Location of transects and historical photographs surveyed at Bramston Reef and Stone Island.

Location	Description	Latitude	Longitude
Bramston Reef	Transect 1	20°03'27.3"	148°15'52.0"
	Transect 2	20°03'27.5"	148°15'53.3"
	Transect 3	20°03'26.8"	148°15'53.1"
	Transect 4	20°03'27.0"	148°15'53.6"
	Elevation transect (start)	20°03'34.4"	148°15'53.8"
	Elevation transect (finish)	20°03'25.3"	148°15'56.3"
	Historical photograph 1	20°03'27.5"	148°15'52.7"
	Historical photograph 2	20°03'25.3"	148°15'56.3"
Stone Island	Transect 1	20°02'35.4"	148°17'12.9"
	Transect 2	20°02'33.1"	148°17'07.7"
	Elevation transect (start)	20°02'31.50"	148°17'8.94"
	Elevation transect (finish)	20°02'39.30"	148°17'14.65"
	Historical photograph 1	20°02'35.2"	148°17'13.9"
	Historical photograph 2	20°02'32.4"	148°17'07.8"

Supplementary Table S2. Descriptions of *in situ* dead coral colonies sampled from Bramston Reef and Stone Island for U-Th dating.

**Sample name**[*]	**Coral sp.**[†]	**Colony height from origin (cm)**	**Section**	**Sample length (cm)**	**Annual growth band width (cm)**[†]				
					**1**	**2**	**3**	**4**	**5**
**Bramston Reef**									
BRS1T1.1	*G. aspera*	43		2.7					
BRS1T1.2	*P. verweyi?*	37		1.8					
BRS1T1.3	*G. aspera*	33		3.5					
BRS1T1.4	*G. aspera*	37	II	2.7	0.64	0.75			
			III	2.8	0.69	0.72			
			IV	3	0.65				
BRS1T2.1	*G. aspera*	31	I	4.1	0.8	0.78	0.83		
BRS1T2.2	*P. verweyi*	49		4.3					
BRS1T2.3	*P. verweyi*	22	I	4.2	0.67	0.65	0.66	0.69	
			II	4.5	0.71	0.61	0.69	0.72	0.71
BRS1T2.4	*P. verweyi*	29	I	4.0	0.56	0.59	0.59	0.6	
			II	4.1	0.79	0.65	0.7	0.73	0.66
BRS1T4.1	*P. verweyi*	68		2.0					
BRS1T4.2	*P. verweyi*	54		3.1					
BRS1T4.3	*P. verweyi*	71		2.6					
BRS1T4.4	*P. verweyi*	61		2.5					
BRS1-R1	*P. verweyi*	66							
BRS1-R2b	*P. verweyi*	62	II	6.4	1.03	0.97	0.97		
			III	6.2	1.02	1.04			
			IV	5.2	1.03	1.04			
BRS1-R3	*P. verweyi*	57	I	3.6	1.35	1.36			
			II	3.6	1.27	1.4			
BRS1-R7c	*P. verweyi*	59		4.5					
BRS1-R8	*P. verweyi*	59							
**Stone Island**									
SIS1T1.1	*Acropora sp.*								
SIS1T1.2	*C. serailia*								
SIS1T1.3	*C. serailia*								
SIS1T1.4	*Acropora sp.*								
SIS1T1.5	*Acropora sp.*								
SIS1T2.1	*Acropora sp.*								
SIS1T2.2	*M. turgescens*								
SIS1T2.3	*M. turgescens*								
SIS1T2.4	*P. lobata*								

[*]For the sample nomenclature, S1 refers to Site 1 where the samples were collected at Bramston Reef and Stone Island. T1-T4 refers to transects 1-4 which were each 20 m in length. The number after the decimal refers to the individual dead coral colony dated from that particular transect.

[†]*G. aspera* = *Goniastrea aspera*, *P. verweyi* = *Platygyra verweyi, Acropora* sp. = *Acropora* (corymbose), *C. serailia* = *Cyphastrea serailia, M. turgescens* = *Montipora turgescens, P. lobata* = *Porites lobata*.

[‡]Growth band width deduced from positive X-radiographs.

Supplementary Table S3. Elemental ratio data for Sr/Ca, Mg/Ca and U/Ca spanning three years of growth for the dead faviid coral Bram_R8 collected from Bramston Reef.

Distance from surface of coral (mm)	Sample name	Mg/Ca (mmol/mol)				Sr/Ca (mmol/mol)				U/Ca (mmol/mol)			
		Run 1	Run 2	Average	sd	Run 1	Run 2	Average	sd	Run 1	Run 2	Average	sd
30	Bram_R8_01	4.32	4.16	4.24	0.11	8.38	8.18	8.28	0.14	1.1120	1.0929	1.1024	0.0067
31	Bram_R8_02	5.425	5.508	5.467	0.058	9.026	8.934	8.980	0.065	1.116	1.070	1.093	0.016
32	Bram_R8_03	4.180	4.241	4.211	0.043	8.9573	8.9558	8.9565	0.0011	1.0729	1.0502	1.0615	0.0081
33	Bram_R8_04	4.3362	4.3467	4.3415	0.0074	8.167	8.044	8.105	0.087	1.0026	0.9971	0.9999	0.0019
34	Bram_R8_05	4.104	4.086	4.095	0.012	8.30	8.14	8.22	0.11	1.0694	1.0535	1.0615	0.0057
35	Bram_R8_06	3.9589	3.9606	3.9597	0.0012	8.289	8.223	8.256	0.047	1.1054	1.0919	1.0987	0.0048
36	Bram_R8_07	3.879	3.857	3.868	0.015	8.288	8.250	8.269	0.027	1.1323	1.1394	1.1359	0.0025
37	Bram_R8_08	3.82537	3.82500	3.82519	0.00026	8.310	8.239	8.274	0.050	1.0976	1.1100	1.1038	0.0044
38	Bram_R8_09	3.8743	3.8627	3.8685	0.0082	9.004	9.020	9.012	0.011	1.1246	1.1196	1.1221	0.0018
39	Bram_R8_10	4.117	4.100	4.109	0.012	8.884	8.935	8.909	0.036	1.1039	1.1293	1.1166	0.0089
40	Bram_R8_11	3.903	3.923	3.913	0.014	8.914	8.994	8.954	0.057	1.11487	1.11696	1.11591	0.00074
41	Bram_R8_12	4.0996	4.1103	4.1050	0.0076	8.177	8.086	8.132	0.064	1.0465	1.0720	1.0593	0.0090
42	Bram_R8_13	4.068	4.095	4.082	0.019	8.100	8.076	8.088	0.017	1.0580	1.0731	1.0655	0.0054
43	Bram_R8_14	4.161	4.136	4.148	0.018	8.162	8.131	8.147	0.022	1.1019	1.1088	1.1053	0.0024
44	Bram_R8_15	3.8397	3.8462	3.8429	0.0046	8.930	9.020	8.975	0.064	1.1578	1.1754	1.1666	0.0062
45	Bram_R8_16	3.802	3.760	3.781	0.029	8.317	8.250	8.283	0.047	1.190	1.148	1.169	0.015
46	Bram_R8_17	3.9380	3.9449	3.9415	0.0049	8.287	8.271	8.279	0.011	1.1119	1.0919	1.1019	0.0071
47	Bram_R8_18	3.9650	3.9768	3.9709	0.0083	8.305	8.231	8.268	0.053	1.112	1.079	1.096	0.012
48	Bram_R8_19	4.059	4.043	4.051	0.011	8.151	8.167	8.159	0.011	1.0448	1.0485	1.0467	0.0013
49	Bram_R8_20	4.026	4.003	4.014	0.016	8.233	8.184	8.209	0.035	1.0840	1.0767	1.0804	0.0026
50	Bram_R8_21	4.187	4.216	4.201	0.021	8.085	8.069	8.077	0.011	1.0379	1.0274	1.0326	0.0037
51	Bram_R8_22	4.122	4.036	4.079	0.061	8.166	8.070	8.118	0.068	1.0521	1.0591	1.0556	0.0025
52	Bram_R8_23	3.94420	3.94447	3.94434	0.00019	8.166	8.151	8.159	0.011	1.0784	1.0877	1.0830	0.0033
53	Bram_R8_24	3.7582	3.7537	3.7559	0.0032	8.935	8.983	8.959	0.034	1.165	1.119	1.142	0.016
54	Bram_R8_25	3.6092	3.6010	3.6051	0.0058	9.089	9.012	9.051	0.054	1.213	1.186	1.200	0.010

Supplementary Table S4. MC-ICP-MS ^{230}Th ages of 26 dead *in situ* coral samples collected from Bramston Reef and Stone Island, inshore GBR.

Sample name*	Sample weight (g)	U (ppm)	^{232}Th (ppb)	(^{230}Th/^{232}Th) meas	^{230}Th/^{238}U	δ^{234}U†	Uncorr. ^{230}Th age (a)	Corr. ^{230}Th age (a)‡	Date of chemistry	Corr. ^{230}Th age (AD)	Sampling distance from surface of colony	No. of annual growth bands above sampling location§	Surface age (AD)¶
**Bramston Reef**													
BRS1T1.1	0.40261	2.8084 ± 0.0032	1.8722 ± 0.0014	3.285 ± 0.044	0.000722 ± 0.000010	145.9 ± 1.0	68.7 ± 0.9	48.9 ± 4.1	2013.7	1964.8 ± 4.1	2.5	3.4 ± 1.7	1968.2 ± 4.4
BRS1T1.2	0.30054	2.8172 ± 0.0015	2.1168 ± 0.0027	2.247 ± 0.071	0.000557 ± 0.000018	147.3 ± 1.4	52.9 ± 1.7	31.0 ± 4.8	2013.7	1982.7 ± 4.8	2.0	2.3 ± 1.1	1985.0 ± 4.9
BRS1T1.3	0.30212	2.8908 ± 0.0030	0.7956 ± 0.0018	4.29 ± 0.12	0.000390 ± 0.000011	144.9 ± 2.0	37.1 ± 1.0	27.3 ± 2.2	2013.7	1986.4 ± 2.2	4.0	5.5 ± 2.7	1991.8 ± 3.5
BRS1T1.4	0.51308	2.6187 ± 0.0027	0.35485 ± 0.00048	7.86 ± 0.15	0.0003508 ± 0.0000068	145.8 ± 2.2	33.4 ± 0.6	26.9 ± 1.5	2013.7	1986.8 ± 1.5	4.0	3.0 ± 0.5^	1989.8 ± 1.5
BRS1T2.1	0.39181	2.6123 ± 0.0011	0.7921 ± 0.0025	18.43 ± 0.18	0.001841 ± 0.000017	145.4 ± 1.3	175.4 ± 1.7	164.7 ± 2.7	2013.7	1849.0 ± 2.7	5.0	4.0 ± 0.5^	1853.0 ± 2.8
BRS1T2.2	0.40992	2.5250 ± 0.0021	0.5195 ± 0.0010	4.98 ± 0.13	0.0003374 ± 0.0000085	145.8 ± 1.8	32.1 ± 0.8	23.7 ± 1.9	2013.7	1990.0 ± 1.9	3.0	3.4 ± 1.7	1993.4 ± 2.6
BRS1T2.3	0.54730	2.4111 ± 0.0034	0.9026 ± 0.0016	3.245 ± 0.071	0.0004004 ± 0.0000088	142.5 ± 2.4	38.2 ± 0.8	25.3 ± 2.7	2013.7	1988.4 ± 2.7	2.0	2.0 ± 0.5^	1990.4 ± 2.8
BRS1T2.4	0.47800	2.7352 ± 0.0032	0.53839 ± 0.00071	5.621 ± 0.090	0.0003646 ± 0.0000058	142.8 ± 1.9	34.8 ± 0.6	26.8 ± 3.0	2013.7	1986.9 ± 1.7	3.0	2.0 ± 2.0	1988.9 ± 2.6
BRS1T4.1	0.51990	2.4328 ± 0.0026	0.30247 ± 0.00036	8.14 ± 0.19	0.0003334 ± 0.0000078	143.0 ± 2.9	31.8 ± 0.7	25.3 ± 1.5	2013.7	1988.4 ± 1.5	4.0	4.6 ± 2.3	1992.9 ± 2.7
BRS1T4.2	0.31033	2.5255 ± 0.0020	1.1116 ± 0.0017	2.977 ± 0.054	0.0004318 ± 0.0000077	143.7 ± 1.5	41.2 ± 0.7	26.8 ± 3.0	2013.7	1986.9 ± 3.0	2.0	2.3 ± 1.1	1989.2 ± 3.2
BRS1T4.3	0.25415	2.5062 ± 0.0021	0.55358 ± 0.00081	5.19 ± 0.16	0.000378 ± 0.000011	145.4 ± 1.2	36.0 ± 1.1	27.2 ± 2.1	2013.7	1986.5 ± 2.1	2.0	2.3 ± 1.1	1988.8 ± 2.4
BRS1T4.4	0.37315	2.7288 ± 0.0033	3.3781 ± 0.0084	1.181 ± 0.016	0.0004820 ± 0.0000066	144.5 ± 1.8	45.9 ± 0.6	11.5 ± 7.0	2013.7	2002.2 ± 7.0	3.0	3.4 ± 1.7	2005.6 ± 7.2
BRS1-R1	0.15013	2.4937 ± 0.0013	1.7457 ± 0.0018	2.319 ± 0.084	0.000535 ± 0.000019	148.6 ± 1.5	50.8 ± 1.8	29.9 ± 4.7	2013.4	1983.6 ± 4.7	3.5	4.0 ± 2.0	1987.6 ± 5.1
BRS1-R2b	0.54858	2.6554 ± 0.0048	0.40610 ± 0.00083	6.44 ± 0.12	0.0003247 ± 0.0000061	144.4 ± 1.6	30.9 ± 0.6	24.0 ± 1.5	2013.7	1989.7 ± 1.5	5.0	2.3 ± 2.3	1991.9 ± 2.7
BRS1-R3	0.15002	2.4498 ± 0.0021	4.5719 ± 0.0065	1.251 ± 0.023	0.000769 ± 0.000014	147.0 ± 1.1	73.1 ± 1.4	22.5 ± 10	2013.4	1991 ± 10	3.0	2.0 ± 0.5^	1993 ± 10
BRS1-R7	0.51373	2.4104 ± 0.0016	0.8804 ± 0.0012	3.229 ± 0.055	0.0003888 ± 0.0000066	147.8 ± 0.7	36.9 ± 0.6	24.3 ± 2.6	2013.7	1989.4 ± 2.6	4.0	4.6 ± 2.3	1993.9 ± 3.5
BRS1-R8	0.14999	2.5433 ± 0.0013	1.9881 ± 0.0043	2.324 ± 0.034	0.0005988 ± 0.0000087	149.9 ± 1.1	56.8 ± 0.8	33.9 ± 4.7	2013.4	1979.6 ± 4.7	5.0	5.0 ± 0.5^	1984.6 ± 4.7
**Stone Island**													
SIS1T1.1	0.15739	3.7263 ± 0.0021	1.7355 ± 0.0028	1.616 ± 0.068	0.000248 ± 0.000010	144.9 ± 1.5	23.6 ± 1.0	9.7 ± 3.1	2015.42	2005.7 ± 3.1	2.0	0.7 ± 0.5	2006.4 ± 3.1
SIS1T1.2	0.15482	2.76162 ± 0.00074	2.3766 ± 0.0039	2.373 ± 0.079	0.000673 ± 0.000022	148.5 ± 1.7	63.9 ± 2.1	39.4 ± 5.5	2015.42	1976.1 ± 5.5	1.0	1.4 ± 1.0	1977.5 ± 5.6
SIS1T1.3	0.16503	2.46926 ± 0.00073	1.1446 ± 0.0022	1.707 ± 0.067	0.000261 ± 0.000010	147.9 ± 1.3	24.8 ± 1.0	9.9 ± 3.2	2015.42	2005.5 ± 3.2	1.0	1.4 ± 1.0	2006.9 ± 3.4
SIS1T1.4	0.18559	3.3649 ± 0.0020	1.3776 ± 0.0029	1.694 ± 0.086	0.000229 ± 0.000012	146.8 ± 1.1	21.7 ± 1.1	9.1 ± 2.9	2015.42	2006.3 ± 2.9	3.0	1.0 ± 0.5	2007.3 ± 2.9
SIS1T1.5	0.14746	3.2291 ± 0.0014	3.6686 ± 0.0072	2.366 ± 0.053	0.000886 ± 0.000020	147.2 ± 0.9	84.2 ± 1.9	53.0 ± 6.6	2015.42	1962.4 ± 6.6	5.0	1.7 ± 1.0	1964.1 ± 6.7
SIS1T2.1	0.15828	3.3910 ± 0.0015	0.7003 ± 0.0019	2.709 ± 0.091	0.0001844 ± 0.0000062	146.8 ± 1.1	17.5 ± 0.6	10.0 ± 1.6	2015.42	2005.4 ± 1.6	7.0	2.3 ± 1.5	2007.7 ± 2.2
SIS1T2.2	0.06997	3.5284 ± 0.0025	23.427 ± 0.060	33.09 ± 0.14	0.072410 ± 0.000236	147.9 ± 2.2	7116 ± 28	6945 ± 44	2015.42	-4930 ± 44	5.0		-4930 ± 44
SIS1T2.3	0.1495	3.6232 ± 0.0017	7.174 ± 0.011	1.569 ± 0.031	0.001024 ± 0.000020	148.0 ± 1.1	97.3 ± 1.9	45 ± 11	2015.42	1970 ± 11	3.0		1970 ± 11
SIS1T2.4	0.15355	3.0997 ± 0.0018	0.9855 ± 0.0037	650.8 ± 3.5	0.068194 ± 0.000267	147.3 ± 1.5	6690 ± 29	6680 ± 29	2015.42	-4664 ± 29	3.0	2.5 ± 1.5	-4661.5 ± 29

Ratios in parentheses are activity ratios calculated from atomic ratios using decay constants of Cheng *et al.*[15]. All values have been corrected for laboratory procedural blanks. All errors reported as 2σ. Uncorrected ^{230}Th age (a) was calculated using Isoplot/EX 3.0 program[16], where *a* denotes year.

*For the sample nomenclature, S1 refers to Site 1 where the samples were collected at Bramston Reef and Stone Island. T1-T4 refers to transects 1-4 which were each 20 m in length. The number after the decimal refers to the individual dead coral colony dated from that particular transect.

†$\delta^{234}U = [(^{234}U/^{238}U)-1]\times 1000$

‡^{230}Th ages corrected using a model two-component correction value based on the equation from Clark *et al.*[17]:

$$\left(\frac{^{230}Th}{^{232}Th}\right)_{mix} = \left(\left(\frac{^{232}Th_{live}}{^{232}Th_{dead}}\right)\times\left(\frac{^{230}Th}{^{232}Th}\right)_{live}\right)+\left(\left(\frac{^{232}Th_{dead}-{}^{232}Th_{live}}{^{232}Th_{dead}}\right)\times\left(\frac{^{230}Th}{^{232}Th}\right)_{sed}\right)$$

where $^{232}Th_{dead}$ is the measured ^{232}Th value (ppb) in the non-living coral sample. ^{232}Thlive is the mean measured ^{232}Th value (ppb) and $^{230}Th/^{232}Th_{live}$ represents or approximates the isotopic composition of the hydrogenous component in the dead coral skeleton with an atomic value of $5.76 \times 10^{-6} \pm 20\%$ (which corresponds to an activity value of 1.066 ± 20%) based on live *Porites* corals collected from the GBR[18]. $^{230}Th/^{232}Th_{sed}$ is the detrital component represented by a mean atomic value of $4.38 \times 10^{-6} \pm 20\%$ (which corresponds to an activity value of 0.81 ± 20%) from isochron derived initial ^{230}Th/^{232}Th values obtained from two dead faviid coral skeletons.

§For samples where no X-rays were available or where annual density banding was ambiguous, the number of potential annual growth bands above the sampling location was calculated by dividing the sampling distance from the top of the colony (cm) by:

i. An average linear extension rate of 7.3 ± 0.5 mm yr^{-1} (1sd) [based on the length of annual density bands (N=8) observed in X-rays taken of two dead *G. aspera* colonies collected in this study] for *G. aspera* colonies

ii. An average linear extension rate of 8.8 ± 2.3 mm yr^{-1} (1sd) [based on the length of annual density bands (N=37) observed in X-rays taken of five dead *P. verweyi* colonies collected in this study] for *P. verweyi* colonies.
iii. An average linear extension rate of ~30 mm yr^{-1} for corymbose *Acropora* spp. colonies[19].
iv. An average linear extension rate of ~12 mm yr-1 for massive *Porites* spp. colonies[19]
v. An average linear extension rate of 7.2 mm yr^{-1} (range 6.6-8.9 mm yr^{-1}) for *C. seralia*[20]
(Average linear extension rate for *M. turgescens* is unknown. The large ^{230}Th age error for the two *M. turgescens* colonies sampled is likely to encompass any age uncertainty associated with sampling location).

Each uncertain layer was assigned a ± 0.5 year uncertainty similar to Rasmussen *et al.*[21] and Clark *et al.*[18]. ^ denotes samples where annual density banding above sampling location was clear.

¶Surface age = Corrected ^{230}Th growth band age + number of growth bands above sampling location.

Surface age error calculated using addition rule for error propagation:

$$\Delta a = \sqrt{(\Delta b)^2 + (\Delta c)^2}$$

where *Δa* = surface age error (years), *Δb* = 2σ ^{230}Th age error (years), *Δc* = uncertainty associated with annual density band counting (years)

Supplementary Table S5. History of cyclones passing within 100 km and 200km of Bowen, Queensland, from 1954 to 2012 AD*.

Year	Month	Cyclone (100 km)	Cyclone (200 km)	Category
2011	23-31 Jan	Anthony		2
2010	12-21 March	Ului		3
2003	1-12 Mar	Erica		3
2000	1-2 Apr		Tessie	2
1997	6-23 Mar		Justin	3
	23-24 Feb		Ita	1
1996	26-29 Jan	Celeste		3
1990	18-27 Dec		Joy	4
1989	16-26 Mar	Ivor		3
	1-5 Apr	Aivu		4
1988	21 Feb - 1 Mar	Charlie		2
1985	18-24 Feb		Pierre	1
1980	2-8 Jan	Paul		2
1979	12 Feb - 4 Mar	Kerry		
	8-11 Jan	Gordon		3
1977	6-10 Mar	Otto		2
	29-31 Jan	Keith		1
1976	25-28 Apr		Watorea	
	3-6 Mar	Dawn		1
1974	8-11 Feb		Yvonne	1
	17-21 Jan	Vera		2
1971	10-16 Feb	Gertie		
1970	2-18 Jan	Ada		4
1964	30 Nov - 8 Dec		Flora	
1961	2-6 Jan	Unnamed #3		
1959	24-31 Dec		Unnamed #2	
	11-17 Feb	Unnamed #8		
1958	31 Mar - 2 Apr	Unnamed #13		
1956	23 Feb - 11 Mar	Agnes		
	15-27 Jan	Unnamed #5		
1955	1-18 Mar		Unnamed #7	
1954	4-8 Feb	Unnamed #2		

*Source: http://www.bom.gov.au/cyclone/history/

Supplementary Table S6. Summary of recovery rates following major (>50% mortality) disturbance in modern reef flat coral communities

Location	Environment	Disturbance	Pre-disturbance levels of cover	Post-disturbance levels of cover	Recovery rate	Mode	Reference
Thousand Islands, Indonesia	Reef flat	1982/83 bleaching	Average 22-26%	2-3%	In 1998 (**5 yrs later)** coral cover was only ***half*** that of pre-disturbance levels (~11-13%)	*Acropora* recruits returned in 1985 with an increase in diversity and community structure by 1988	[22]
Shiraho Reef, Ryukyus, NW Pacific	Seaward reef crest	1997/98 bleaching	~20-40%	~10-20% cover (41.1% mortality in *Porites*, 55.4% in *Montipora*, 82.4% in *Acropora)*	Recovery of *Montipora* and *Acropora* to pre-disturbance coverage **1-2 yrs later**.	Recovery was accomplished by asexual regrowth of parts of the coral that survived the 1998 bleaching	[23]
Kaneohe Bay, Oahu, Hawaii	Reef flat (actively growing margins)	1965 flood combined with sewage discharge between 1964 and 1979.	1965 event: no data	1965 event: 0% cover	1965 event: Recovery slow or non-existent due to eutrophic conditions, even 14 years later. Reef flat communities rapidly increased in coral coverage and diversity after the outfall was removed in1979, increasing to ~35-70% cover in 1983.	1965 event: Recruitment	[24]
		1987 flood<br><br>(These events were compared to understand recovery under polluted and non-polluted conditions)	~60-70% cover	0% cover (100% mortality along fringing reef and reef flat).	~25-50% cover **5-10 years later**.	Most components of the reef flat community recovered in 1-2 yrs attributed to the regeneration of surviving tissue (*Porites compressa* and *Fungia scutaria*) and some recruitment of *Montipora* and	

						Pocillopora (<1% cover)	
Cocos Island	Lagoon	1876 'dark-coloured and malodorous water'following a cyclone in January 1876 (p. 192)		'…a vast field of blackened and lifeless coral stems,,,' (p. 192)	'…here and there a new branch of Madrepora and Porites' (p. 192) two years after the event.<br><br>'…a lifeless waste…' 12.5 years later (p. 193)<br><br>**Little sign of recovery 30 years later** (between 1905-1906) '…many places…practically no signs of life amongst the dead coral masses…and algae have for most part been the successors of coral life.' (p. 193)		[25]
Snapper Island South, inshore GBR	Shallow reef slope/crest (2-3m below datum)	1996 flood<br><br>1999 cyclone Rona	85-88% in 1994	<15% cover (corals on the reef flat and slope almost all dead. 100% loss of acroporids. Few surviving massive poritids and faviids)<br><br>Dead *in situ* acroporids turned into rubble, poritid and faviids slightly damaged.	After 1996 coral cover remained ~10% before increasing to ~18% in 2005, 39% in 2009 and 50% in 2012 **(13 years**).		[26-28]
Snapper Island North, inshore GBR	Shallow reef slope/crest (2-3m below datum)	1999 cyclone Rona	~68%	~18% (74% decline)	~40% in 2005 (**14 yrs**) increasing to 52% in 2009 **(18 yrs)**		[27,28]
Fitzroy Island West, inshore GBR	Shallow reef slope/crest (2-3m below datum)	1999-2000 COTS outbreak	No data	78% decline in coral cover	41% in 2009 increasing to 45% in 2012 **(12 yrs later)**		[28]
Frankland Group East, inshore GBR	Shallow reef slope/crest (2-3m below datum)	1998 bleaching<br><br>1999-2000 COTS outbreak	~53% cover in 1995<br><br>~30%	~30% cover<br><br>14% (59-66% decline			[27,28]

		Cyclone Larry 2006	~35%	coral cover<br><br>21% (60% decline)	26% in 2009 (**3 years)**		
Frankland Group West		1998 bleaching	~80% in 1995 (mostly *Acropora* and poritids)	~36% (~45% reduction in coral cover)	Increase to ~46% in 1999		[27]
North Barnard Group, inshore GBR	Shallow reef slope/crest (2-3m below datum)	2006 cyclone Larry	~50% in 2005	~3% (95% decline)	7% in 2009		[28]
Dunk Island North, inshore GBR	Shallow reef slope/crest (2-3m below datum)	2006 cyclone Larry	~45% in 2005	~9% (80% decline)	15% in 2009 increasing to 25% in 2010		[28]
Pelorus & Orpheus Island West	Shallow reef slope/crest (2-3m below datum)	1998 bleaching	No data	83% decline	10% in 2009		[28]
Pandora Reef	Shallow reef slope/crest (2-3m below datum)	2000 Cyclone Tessie		78% decline	4% in 2009		[28]
Havannah Island	Shallow reef slope/crest (2-3m below datum)	1998 bleaching<br><br>2000 Cyclone Tessie and COTS		49% decline<br><br>66% decline	19% in 2009		[28]
Dent Island, Whitsundays	Shallow reef slope/crest (2-3m below datum)	1998 bleaching	No data	40% decline	~55% in 2005 **(7 yrs later)** and 54% in 2009		[28]
Daydream Is, Whitsundays	Shallow reef slope/crest (2-3m below datum)	1998 bleaching	No data	40% decline	~35% in 2005 **(7 yrs later**) and 32% in 2009.		[28,29]
Halfway Island	Up to 1.3 m LWD	1991 flood	66%	0% at -0.3m LWD	60% in 2005 **(14 years**)	Recruitment from deeper waters that survived flood	[28,30]
Middle Island	Up to 1.3 m LWD	1991 flood	>83%	0% (100% mortality)	80% in 2005 **(14 years)**	Recruitment from deeper waters that survived flood	[28,30]
Heron Island, offshore GBR	Exposed crest	1972 cyclone (including alteration of physical environment)	~70%	0%	Only 20% cover reached in 1992 **(20 yrs later)**	Recruitment occurred only after the substrate had been eroded away	[31]

Supplementary Movies

Supplementary Movie S1. Underwater video footage of the reef crest and slope environment at Bramston Reef, 31 July 2012. Note the high abundance of macroalgae and patchy distribution of live coral cover. Longer version available from the authors.

Supplementary Movie S2. Underwater video footage of the reef crest and slope environment at Stone Island, 31 July 2012. Note the high abundance of macroalgae and very few living corals. Longer version available from the authors.

Supplementary References

1 Wachenfeld, D. R. Long-term trends in the status of coral reef-flat benthos - The use of historical photographs. (Great Barrier Reef Marine Park Authority, 1997).

2 Hedley, C. The natural destruction of a coral reef.Transactions of the Royal Geographical Society (Queensland) 35-40 (1925).

3 Brodie, J., Ariel, E., Thomas, C., O'Brien, D. & Berry, K. Links between water quality and marine turtle health. 88 (Centre for Tropical Water & Aquatic Ecosystems Research (TropWATER), Townsville, 2014).

4 GHD. Report for Bowen WWTF Upgrades, Planning Study. Report prepared for Whitsunday Regional Council. 101 (2010).

5 Jones, R. J., Muller, J., Haynes, D. & Schreiber, U. Effects of herbicides diuron and atrazine on corals of the Great Barrier Reef, Australia. *Mar. Ecol-Prog. Ser.* **251**, 153-167 (2003).

6 Fabricius, K. A. Effects of terrestrial runoff on the ecology of corals and coral reefs: review and synthesis. *Mar. Pollut. Bull.* **50**, 125-146 (2005).

7 Wooldridge, S. A. Water quality and coral bleaching thresholds: Formalising the linkage for the inshore reefs of the Great Barrier Reef, Australia. *Mar.Pollut. Bull.* **58**, 745-751 (2009).

8 Faithful, J. Water quality in the Whitsundays Rivers. Surface water quality - December 2000 to August 2002, 82 (Australian Centre for Tropical Freshwater Research, Townsville, 2002).

9 Rohde, K., Brodie, J. E. & Faithful, J. W. Fresh and marine water quality in the Mackay Whitsunday region, 2004/05 to 2006/07: The event monitoring component of the integrated monitoring program, Mackay Whitsunday Healthy Waterways, a community-assisted approach. . (Department of Natural Resources and Water, Mackay, 2008).

10 Kroon, F. J. *et al.* River loads of suspended solids, nitrogen, phosphorous and herbicides delivered to the Great Barrier Reef lagoon. *Mar. Pollut. Bull.* **65**, 167-181 (2012).

11 McCulloch, M. T., Gagan, M. K., Mortimer, G. E., Chivas, A. R. & Isdale, P. J. A high-resolution Sr/Ca and δ^{18}O coral record from the Great Barrier Reef, Australia, and the 1982-1983 El Niño. *Geochim. Cosmochim. Acta* **58**, 2747-2754, doi:10.1016/0016-7037(94)90142-2 (1994).

12 Marshall, J. F. & McCulloch, M. T. An assessment of the Sr/Ca ratio in shallow water hermatypic coral as a proxy for sea surface temperature. *Geochim. Cosmochim. Acta* **66**, 3263-3280 (2002).

13 Fallon, S. J., McCulloch, M. T. & Alibert, C. Examining water temperature proxies in *Porites* corals from the Great Barrier Reef: a cross-shelf comparison. *Coral Reefs* **22**, 389-404 (2003).

14 Saville-Kent, W. *The Great Barrier Reef.* (W.H. Allen, 1893).

15 Cheng, H. *et al.* The half-lives of uranium-234 and thorium-230. *Chem. Geol.* **169**, 17-33 (2000).

16 Ludwig, K. R. *Users Manual for Isoplot/Ex version 3.0: A Geochronological Toolkit for Microsoft Excel.* (Berkeley Geochronology Centre Special Publication No.3., 2003).

17 Clark, T. R. *et al.* Testing the precision and accuracy of the U-Th chronometer for dating coral mortality events in the last 100 years. *Quat. Geochronol.* **23**, 35-45 (2014).

18 Clark, T. R. *et al.* Spatial variability of initial ^{230}Th/^{232}Th in modern *Porites* from the inshore region of the Great Barrier Reef. *Geochim. Cosmochim. Acta* **78**, 99-118, doi:10.1016/j.gca.2011.11.032 (2012).

19 Pratchett, M. S. *et al.* Spatial, temporal and taxonomic variation in coral growth - Implications for the structure and function of coral reef ecosystems. *Oceanogr. Mar. Biol.* **53**, 215-295 (2015).

20 Roberts, L. G. & Harriott, V. J. Can environmental records be extracted from coral skeletons from Moreton Bay, Australia, a subtropical, turbid environment? *Coral Reefs* **22**, 517-522 (2003).

21 Rasmussen, S. O. *et al.* A new Greenland ice core chronology for the last glacial termination. *J. Geophys. Res.* **111**, doi:10.1029/2005JD006079, doi:10.1029/2005JD006079 (2006).

22 Brown, B. E. & Suharsono. Damage and recovery of coral reefs affected by El Nino related seawater warming in the Thousand Islands, Indonesia. *Coral Reefs* **8**, 163-170 (1990).

23 Kayanne, H., Harii, S., Ide, Y. & Akimoto, F. Recovery of coral populations after the 1998 bleaching on Shiraho Reef, in the southern Ryukyus, NW Pacific. *Mar. Ecol-Prog. Ser.* **239**, 93-103 (2002).

24 Jokiel, P. L., Hunter, C. L., Taguchi, S. & Watarai, L. Ecological impact of a fresh-water 'reef kill' in Kaneohe Bay, Oahu, Hawaii. *Coral Reefs* **12**, 177-184 (1993).
25 Jones, F. W. *Coral and atolls: A history and description of the Keeling-Cocos Islands, with an account of their fauna and flora, and a discussion of the method of development and transformation of coral structures in general.* (Lovell, Reeve and Co. Ltd., 1912).
26 Ayling, A. M. & Ayling, A. L. The effect of the Daintree River flood plume on Snapper Island coral reefs. (Great Barrier Reef Marine Park Authority, Townsville, 1998).
27 Ayling, A. M. & Ayling, A. L. The Dynamics of Cairns section fringing reefs: part II. (Sea Research, 2000).
28 Thompson, A. *et al.* Reef Rescue Marine Monitoring Program. Report of AIMS Activities - Inshore coral reef monitoring 2012. Report for the Great Barrier Reef Marine Park Authority. 120 (Australian Institute of Marine Science, Townsville, 2013).
29 Sweatman, H. *et al.* Long-term monitoring of the Great Barrier Reef: Status Report No. 8. (Australian Institute of Marine Science, Townsville, 2008).
30 Van Woesik, R. Immediate impact of the January 1991 floods on the coral assemblages of the Keppel Islands. 33 (Great Barrier Reef Marine Park Authority, Townsville, 1991).
31 Connell, J. H., Hughes, T. P. & Wallace, C. C. A 30-year study of coral abundance, recruitment, and disturbance at several scales in space and time. *Ecol. Monogr.* **67**, 461-488 (1997).
